# Protein intake and body weight, fat mass and waist circumference: an umbrella review of systematic reviews for the evidence-based guideline on protein intake of the German Nutrition Society

**DOI:** 10.1007/s00394-023-03220-x

**Published:** 2023-10-04

**Authors:** Sabine Ellinger, Anna M. Amini, Julia Haardt, Andreas Lehmann, Annemarie Schmidt, Heike A. Bischoff-Ferrari, Anette E. Buyken, Anja Kroke, Tilman Kühn, Sandrine Louis, Stefan Lorkowski, Katharina Nimptsch, Matthias B. Schulze, Lukas Schwingshackl, Roswitha Siener, Gabriele I. Stangl, Dorothee Volkert, Armin Zittermann, Bernhard Watzl, Sarah Egert, the German Nutrition Society

**Affiliations:** 1 Institute of Nutritional and Food Science, Human Nutrition, University of Bonn, Meckenheimer Allee 166a, 53115 Bonn, Germany; 2 German Nutrition Society, Bonn, Germany; 3 Department of Aging Medicine and Aging Research, University Hospital and University of Zurich, Zurich, Switzerland; 4 City Hospital Zurich, Zurich, Switzerland; 5 Institute of Nutrition, Consumption and Health, Faculty of Natural Sciences, Paderborn University, Paderborn, Germany; 6 Department of Nutritional, Food and Consumer Sciences, Fulda University of Applied Sciences, Fulda, Germany; 7 The Institute for Global Food Security, Queen’s University Belfast, Belfast, Northern Ireland UK; 8 Faculty of Medicine and University Hospital, Heidelberg Institute of Global Health (HIGH), Heidelberg, Germany; 9 Department of Nutritional Sciences, University of Vienna, Vienna, Austria; 10 Center for Public Health, Medical University of Vienna, Vienna, Austria; 11 Department of Physiology and Biochemistry of Nutrition, Max Rubner-Institut, Karlsruhe, Germany; 12 Institute of Nutritional Sciences, Friedrich Schiller University Jena, Jena, Germany; 13 Competence Cluster for Nutrition, Cardiovascular Health (nutriCARD) Halle-Jena-Leipzig, Jena, Germany; 14 Molecular Epidemiology Research Group, Max Delbrück Center for Molecular Medicine (MDC), Helmholtz Association, Berlin, Germany; 15 Department of Molecular Epidemiology, German Institute of Human Nutrition Potsdam-Rehbruecke, Nuthetal, Germany; 16 Institute of Nutritional Science, University of Potsdam, Potsdam, Germany; 17 German Center for Diabetes Research (DZD), Munich-Neuherberg, Germany; 18 Institute for Evidence in Medicine, Medical Center, Faculty of Medicine, University of Freiburg, Freiburg, Germany; 19 Department of Urology, University Stone Center, University Hospital Bonn, Bonn, Germany; 20 Institute of Agricultural and Nutritional Sciences, Martin Luther University Halle-Wittenberg, Halle (Saale), Germany; 21 Institute for Biomedicine of Aging, Friedrich-Alexander-Universität Erlangen-Nürnberg, Nuremberg, Germany; 22 Clinic for Thoracic and Cardiovascular Surgery, Herz- und Diabeteszentrum Nordrhein-Westfalen, Ruhr University Bochum, Bad Oeynhausen, Germany; 23 Institute of Nutritional and Food Science, Nutritional Physiology, University of Bonn, Bonn, Germany

**Keywords:** Umbrella review, Protein intake, Body weight, Fat mass, Waist circumference

## Abstract

**Purpose:**

This umbrella review aimed to assess whether dietary protein intake with regard to quantitative (higher vs. lower dietary protein intake) and qualitative considerations (total, plant-based or animal-based protein intake) affects body weight (BW), fat mass (FM) and waist circumference (WC).

**Methods:**

A systematic literature search was conducted in PubMed, Embase and Cochrane Database of Systematic Reviews for systematic reviews (SRs) with and without meta-analyses of prospective studies published between 04 October 2007 and 04 January 2022. Methodological quality and outcome-specific certainty of evidence of the retrieved SRs were assessed by using AMSTAR 2 and NutriGrade, respectively, in order to rate the overall certainty of evidence using predefined criteria.

**Results:**

Thirty-three SRs were included in this umbrella review; 29 were based on randomised controlled trials, a few included cohort studies. In studies without energy restriction, a high-protein diet did not modulate BW, FM and WC in adults in general (all “possible” evidence); for older adults, overall certainty of evidence was “insufficient” for all parameters. Under hypoenergetic diets, a high-protein diet mostly decreased BW and FM, but evidence was “insufficient” due to low methodological quality. Evidence regarding an influence of the protein type on BW, FM and WC was “insufficient”.

**Conclusion:**

“Possible” evidence exists that the amount of protein does not affect BW, FM and WC in adults under isoenergetic conditions. Its impact on the reduction in BW and FM under hypoenergetic conditions remains unclear; evidence for an influence of protein type on BW, FM and WC is “insufficient”.

**Supplementary Information:**

The online version contains supplementary material available at 10.1007/s00394-023-03220-x.

## Introduction

Obesity is a chronic disease worldwide; its prevalence almost tripled between 1975 and 2016 [1]. Android obesity is of great concern due to the increased risk of obesity-associated diseases, such as type 2 diabetes mellitus, hypertension, cardiovascular diseases, and many types of cancer [2–4]. To prevent obesity, energy intake has to be adapted to the individual energy requirement. However, this is challenging as the availability of energy-dense foods [5] and physical inactivity have increased [6].

Diets rich in protein may be beneficial for the prevention and treatment of obesity as protein exerts a higher diet-induced thermogenesis than carbohydrates (CHO) and fat. Moreover, protein can maximise lean body mass retention during weight loss, which in turn may counteract a decrease in resting energy expenditure [7]. Furthermore, dietary protein stimulates the release of intestinal peptide hormones (e.g., glucagon like peptide-1, peptide YY) with anorexigenic properties, which may increase satiety, thereby reducing food consumption and energy intake [7]. Overall, high protein intake might favour a negative energy balance by increasing energy expenditure and decreasing energy intake; both might contribute to normalisation of body weight (BW) [7].

In addition, protein quality (digestibility, content of indispensable/essential amino acids [EAA], particularly leucine as key stimulus in muscle protein synthesis, availability of amino acids for muscle protein synthesis) may also be relevant for the prevention and treatment of obesity [8]. For example, whey protein has a higher protein quality (rapid digestion, high content of EAA) than casein. Moreover, amino acids from whey protein and casein are less catabolised than soya protein, thus increasing their availability for muscle protein synthesis [8]. Soya protein is digested rapidly, but contains less EAA than whey protein. Therefore, whey protein may be beneficial during weight loss since a higher preservation of lean body mass (or muscle mass) can be expected [8]. Moreover, whey protein and casein have higher effects on satiety than other proteins, which could promote weight loss by a stronger suppression of hunger sensations [8, 9].

International recommendations on protein intake are around 0.8 g/kg BW/day for healthy adults [10]. This corresponds to a mean daily intake of 11% of energy (EN%) for an adult with a reference BW of 70 kg and a total energy requirement of 2200 kcal/day. The recommendations on protein intake apply to people with normal weight; for people with under- or overweight or obesity, it is recommended to adjust the BW to the reference weight. In European countries, average protein intake of adults ranged from 67 to 114 g/day for men and 59 to 102 g/day for women, which corresponds to an average intake of 0.8–1.25 g/kg BW/day or 12–20 EN% [11].

The German Nutrition Society is currently developing an evidence-based guideline for protein intake regarding the impact of protein amount and type on several outcomes in the general adult population, namely bone health [12], kidney health [13], blood pressure, cancer, cardiovascular diseases, muscle health, type 2 diabetes mellitus and BW and related outcomes [14]. The current manuscript focuses on the latter. The collection of evidence for each outcome will form the basis for the overall conclusion of the guideline [14].

The key question behind this umbrella review was to assess the overall certainty of evidence whether dietary protein intake with regard to quantitative (higher vs. lower dietary protein intake) and qualitative (total, plant-based or animal-based protein intake) considerations affects BW, fat mass (FM) and waist circumference (WC).

## Methods

We conducted an umbrella review (PROSPERO: CRD42018082395) following the methodology published by Kroke et al. [14]. Two authors each independently conducted the literature search, selection of systematic reviews (SRs), data extraction, assessment of methodological quality and outcome-specific certainty of evidence (AMA, JH, AL, AS), as well as the grading of the overall certainty of evidence (SE, SEg). Any disagreements were resolved by discussion and consensus [14].

### Data sources and searches

The systematic literature search was performed in PubMed, Embase and Cochrane Database of Systematic Reviews for SRs with or without meta-analyses (MA) published between 04.10.2007 and 04.01.2022. The date of 10/2007 originates from the decision to cover a 10-year period, i.e. the initial database search was conducted in 10/2017, and the last update in 01/2022. The search strategies are presented in Supplementary Material 1. In addition to the database search, the reference lists of the included SRs were reviewed.

### Selection of systematic reviews

Titles and/or abstracts of retrieved records were screened according to the pre-defined inclusion/exclusion criteria to identify potentially eligible publications. The full texts of potentially relevant publications were assessed for eligibility. It was tolerated that some of the primary studies were incorporated more than once into different SRs, the overlap of primary studies was documented and the percentage of overlapping was assessed by calculating the corrected cover area according to Pieper et al. [15].

Publications were included if they met the following criteria: (i) evaluated the association between protein intake and BW, FM and WC in the general adult population including older adults and recreational athletes, (ii) SR with or without MA of prospective studies in humans, i.e. randomised controlled trials (RCTs), prospective cohort studies, case-cohort studies or nested case–control studies. Inclusion of case–control studies was tolerated if another study type was predominant, (iii) manuscript was written in English or German.

Exclusion criteria were as follows: (i) study populations consisted exclusively of children, pregnant and/or lactating women and/or top athletes, (ii) not investigating the specific effect of protein, (iii) not investigating relevant protein–outcome relationships, (iv) conference proceedings or abstracts, (v) individual studies (RCTs, cohort studies, other primary studies), and (vi) umbrella reviews.

### Data extraction

The following data from each included SR were extracted into a standardised form: the first author’s surname, year of publication, study type, duration range of primary studies, study population, intervention/exposure(s), outcome(s), effect estimate(s) including 95% confidence interval, *P*-value(s) and heterogeneity estimate(s). In case of missing data, corresponding authors were contacted.

### Assessment of methodological quality and outcome-specific certainty of evidence

The methodological quality of each retrieved SR was assessed by a modified version of the “A Measurement Tool to Assess Systematic Reviews 2” tool (AMSTAR 2) [16] (Supplementary Material 2). SRs were rated on a scale from “high” to “critically low” quality. SRs graded as “critically low” by AMSTAR 2 were excluded from the current work.

The NutriGrade scoring tool was used to rate the outcome-specific certainty of evidence of included SRs [17] by means of a numerical scoring system as high, moderate, low, or very low (Supplementary Material 3). The NutriGrade scoring tool was modified for the assessment of SRs without MA, as described by Kroke et al. [14]. If an SR reported more than one relevant outcome, each outcome-specific certainty of evidence was assessed separately.

### Grading of the overall certainty of the evidence

After summarising the available evidence, two authors (SE and SEg) graded the overall certainty of evidence based on the criteria outlined in our protocol [14] and in Table 1. This rating was double-checked by staff members of the German Nutrition Society (AMA and JH) and thereafter reviewed and approved by all co-authors.

**Table 1:** Grading the overall certainty of evidence according to methodological quality, outcome-specific certainty of evidence, biological plausibility and consistency of results, and definition of the overall certainty of evidence in a modified form according to the GRADE approach [14, 56]

Overall certainty of evidence	Underlying criteria	Definition/explanation
Convincing	• At least one SR with or without MA of prospective studies available • If more than one SR with or without MA are available: all overall results must be consistent.^1^ • In case of a positive or negative association, biological plausibility is given • All included SRs with or without MA must reach at least a “moderate” outcome-specific certainty of evidence^2^; in addition, all included SRs must reach at least a methodological quality^3^ of “moderate”	There is high level of confidence that the true effect lies close to that of the estimate(s) of the effect
Probable	• At least one SR with or without MA of prospective studies available • If more than one SR with or without MA are available, the majority of overall results must be consistent.^1^ • In case of a positive or negative association, biological plausibility is given • The majority^4^ of included SRs with or without MA must have reached at least a “moderate” outcome-specific certainty of evidence^2^; in addition, all included SRs must reach at least a methodological quality^3^ of “moderate”	There is moderate confidence in the effect estimate(s): The true effect is likely to be close to the estimate of the effect, but there is a possibility that it is substantially different
Possible	• At least one SR with or without MA of prospective studies available • If more than one SR with or without MA are available, the majority of overall results must be consistent.^1^ • In case of a positive or negative association, biological plausibility is given • The majority^4^ of included SRs with or without MA must reach at least a “low” outcome-specific certainty of evidence^2^; in addition, the majority^4^ of all included SRs must reach at least a methodological quality^3^ of “moderate”	Confidence in the effect estimate(s) is limited: The true effect may be substantially different from the estimate of the effect
Insufficient	• No SR is available *OR* • The majority^4^ of included SRs with or without MA reach a “very low” outcome-specific certainty of evidence^2^; in addition, the majority of all included SRs reach a methodological quality^3^ of “low”	There is very little confidence in the effect estimate(s): The true effect is likely to be substantially different from the estimate of effect

*MA* meta-analysis, *SR* systematic review

^1^Consistent: overall results of the SR have to be consistently either risk reducing or risk elevating or consistently showing no risk association

^2^Outcome-specific certainty of evidence refers to the NutriGrade rating

^3^Methodological quality refers the AMSTAR 2 rating; SRs graded as “critically low” by AMSTAR 2 are not considered

^4^Majority: > 50% of the included SRs

## Results

**Fig. 1:**
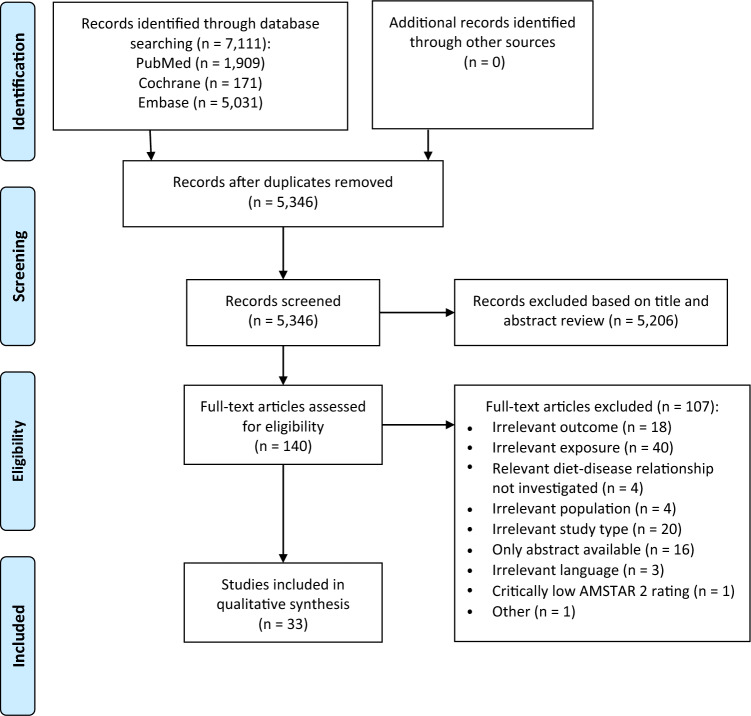
Flow diagram

The process of study selection is outlined in Fig. 1. The literature search identified 7111 potentially relevant records. After removal of duplicates, 5206 records were excluded by screening on the basis of title and/or abstract. Thereafter, 107 records were excluded after assessing the full text. A total of 33 SRs were finally included in the present umbrella review [18–50]. These SRs were published between 07/2009 and 03/2022. A list of excluded records after full-text assessment including justifications for exclusion is provided in Supplementary Material 4.

### Study characteristics

Among these 33 SRs, 29 included solely RCTs [18–29, 31, 32, 35–42, 44–50]. One SR considered solely cohort studies [30] and three SRs additionally addressed RCTs [33, 34, 43]. In total, 26 SRs conducted an MA [20–29, 32, 35–42, 44–50] and seven SRs were without MA [18, 19, 30, 31, 33, 34, 43]. One SR with MA analysed dose–response relationships [35]. Some of the primary studies were incorporated more than once into different SRs, but overall there was only a “slight” overlap of primary studies of 2.2%, determined as corrected covered area according to Pieper et al. [15], see Supplementary Material 5. BW was addressed in 29 SRs [18–20, 22, 23, 26–35, 37–50], FM in 21 SRs [18, 23–25, 27, 29, 32–34, 36–43, 45, 46, 49, 50] and WC in 13 SRs [18, 21, 30, 32–35, 40, 42, 43, 48–50].

Most SRs investigated the effect/association of total protein [18, 21–42, 44–50], and five compared different types of protein (e.g., soya vs. whey protein) [19, 20, 25, 40, 43]. Protein intake differed highly between the SRs, but the amount in the control group reached at least the recommended protein intake for adults (0.8 g/kg BW/day for adults < 65 years, 1.0 g/kg BW/day for adults > 65 years, which corresponds to an intake about 10% EN under isoenergetic conditions) (Table 2). Dietary intervention of included RCTs ranged from two to 208 weeks; the duration of included cohort studies ranged from one to seven years (Table 2). In three SRs, the study durations of included studies were not mentioned or remained unclear [33, 34, 48]. Six SRs focused on the effects of protein and/or different protein types in older adults [22, 23, 28, 29, 36, 41]. All SRs except one [27] were based on studies with men and women. The included SRs contained healthy participants [18, 25, 27, 33, 45] and subjects with risk factors of cardiometabolic diseases [20, 21, 23, 26, 29, 36, 42, 48], one SR focused on nursing home residents [22] and another on older adults with frailty [29]. In most SRs, specific restrictions on the participants’ health status were lacking [19, 22, 24, 28, 30–32, 34, 35, 37–41, 43, 44, 46, 47, 49, 50].

**Table 2:** Characteristics of the included systematic reviews

Author, year	Study type, study duration/follow up	Study population	Exposition	Outcome	Effect estimates	Heterogeneity estimators	NutriGrade rating	AMSTAR 2 rating
Damaghi 2022 [46]	- SR with MA of RCTs - RCTs published before 08/20 - Study duration: 2–36 wk	- Both sexes, - With or without overweight or obesity, - Age range: 18–65 y	Soya protein (22–70 g/d) and/or whey protein (22–56 g/d) vs. maltodextrin		Pooled MD (95% CI), random effect:			High
		*n* = 430, 6 RCTs	Soya or whey protein vs. maltodextrin	BW	− 0.63 kg (− 1.24, − 0.02) *P* NS	*I*^2^ = 10% *P* = 0.35	Moderate	
		6 RCTs	Soya protein vs. maltodextrin	BW	− 0.58 kg (− 1.32, 0.16) *P* NS	*I*^2^ = 0% *P* = 0.90	Moderate	
		6 RCTs	Whey protein vs. maltodextrin	BW	− 0.46 kg (− 1.92, 1.00) *P* NS	*I*^2^ = 51% *P* = 0.07	Moderate	
		*n* = 488, 7 RCTs	Soya or whey protein vs. maltodextrin	FM (kg)	− 0.03 kg (− 0.65, 0.60) *P* NS	*I*^2^ = 0% *P* = 0.99	Moderate	
		*n* = 91, 2 RCTs	Soya or whey protein vs. maltodextrin	FM (%)	0.48% (− 0.37, 1.33) *P* NS	*I*^2^ = 0% *P* = 0.69	Low	
		7 RCTs	Soya protein vs. maltodextrin	FM (kg)	0.09 kg (− 0.88, 1.07) *P* NS	*I*^2^ = 0% *P* = 0.87	Moderate	
		2 RCTs	Soya protein vs. maltodextrin	FM (%)	0.81% (− 0.44, 2.07) *P* NS	*I*^2^ = 0% *P* = 0.76	Low	
		6 RCTs	Whey protein vs. maltodextrin	FM (kg)	− 0.11 kg (− 0.92, 0.70) *P* NS	*I*^2^ = 0% *P* = 0.99	Moderate	
		2 RCTs	Whey protein vs. maltodextrin	FM (%)	0.20% (− 0.96, 1.36) *P* NS	*I*^2^ = 0% *P* = 0.35	Low	
Zhang 2022 [50]	- SR with MA of weight-loss RCTs - RCTs published before 07/19 - Study duration: 12 wk–12 mo	- Both sexes, - Mean age: 37.5–49.8 y, - Mean BMI: 28.5–36.3 kg/m^2^, **-** No information on health status	High-protein meal replacement (15–35 EN% protein) vs. control diet (10–20 EN% protein)		Pooled SMD (95% CI), random effect:			Moderate
		*n* = 814, 8 RCTs		BW	− 0.24 (− 0.37, − 0.10) *P* sig	*I*^2^ = 0% *P* = 0.53	Low	
		*n* = 452, 5 RCTs		FM	− 0.37 (− 0.65, − 0.09) *P* sig	*I*^2^ = 54% *P* = 0.07	Low	
		*n* = 599, 6 RCTs		WC	− 0.24 (− 0.56, 0.09) *P* NS	*I*^2^ = 75% *P* = 0.01	Low	
Hansen 2021 [47]	- SR with MA of RCTs - RCTs published before 09/21 - Study duration: 8–104 wk	- Both sexes, - With overweight or obesity, - Mean age: 22–62 y, - Mean BMI: 29.4–45.6 kg/m^2^, - With or without comorbidities		BW	Pooled MD (95% CI), random effect:			Moderate
		*n* = 4785, 41 RCTs	Increased protein intake (18–59 EN%) vs. controls (digestible CHO, fibre, fat or no supplementation, no placebo used)		− 1.56 kg (− 1.96, − 1.16) *P* NP	*I*^2^ = 56% *P* = 0.01	Moderate	
		*n* = 4159, 35 RCTs	Protein vs. digestible CHO		− 1.66 kg (− 2.11, − 1.21) *P* NP	*I*^2^ = 59% *P* = 0.01	Moderate	
		*n* = 208, 2 RCTs	Protein vs. fibre		− 1.18 kg (− 2.18, − 0.18) *P* NP	*I*^2^ = 0% *P* = 0.72	Very low	
		*n* = 150, 2 RCTs	Protein vs. fat		− 0.28 kg (− 1.83, 1.27) *P* NP	*I*^2^ = 47% *P* = 0.17	Very low	
		*n* = 268, 2 RCTs	Protein supplementation vs. no supplementation (no placebo used)		− 1.90 kg (− 3.19, − 0.61) *P* NP	*I*^2^ = 0% *P* = 0.36	Very low	
Mohammadifard 2021 [48]	- SR with MA of RCTs - RCTs published before 08/20 - Study duration: 8–12 wk	*n* = 145, 3 RCTs - Both sexes, - All with MS, - Age range: 45–75 y,			Pooled MD (95% CI), fixed effect:			Low
			Soya protein	BW	− 0.08 kg (− 0.37, 0.21)	*I*^2^ = 43%	Very low	
			Soya protein	WC	− 0.19 cm (− 0.48, 0.10)	*I*^2^ = 0%	Very low	
Vogtschmidt 2021 [49]	- SR with MA of RCTs - RCTs published before 11/20 - Study duration: 4–156 wk	- Both sexes, - Mean age: 23–70 y, - Mean BMI: 24–39 kg/m^2^, - Healthy or with overweight, obesity, hypertension, hyperinsulinaemia, hyperlipidaemia, MS, PCOS or diabetes	Higher (20–45 EN%) vs. lower protein (10–23 EN%) diets		Pooled SMD/MD (95% CI), random effect:			High
		*n* = 3346, 48 RCTs		BW	SMD: − 0.13 (− 0.23, − 0.03) *P* sig	*I*^2^ = 38% *P* = 0.01	Low	
		*n* = 3346, 48 RCTs		BW	MD: − 0.64 kg (− 1.12, − 0.17) *P* sig	*I*^2^ = 53% *P* = 0.01	Low	
		*n* = 774, 3 RCTs		BW regain	SMD: − 0.18 (− 0.32, − 0.04) *P* NP	*I*^2^ = 0% *P* = 0.6	Very low	
		*n* = 2580, 35 RCTs		FM	SMD: − 0.14 (− 0.24, − 0.04) *P* sig	*I*^2^ = 28% *P* = 0.1	Low	
		*n* = 2580, 35 RCTs		FM	MD: − 0.55 kg (− 0.92, − 0.17) *P* sig	*I*^2^ = 28% *P* = 0.1	Low	
		*n* = 2669, 26 RCTs		WC	SMD: − 0.11 (− 0.23, 0.01) *P* NS	*I*^2^ = 44% *P* = 0.01	Low	
Blair 2020 [18]	- SR without MA of RCTs - RCTs published before 03/20 - Study duration: 2 wk–18 mo	*n* = 638, 10 RCTs, - Both sexes, - Mean age: 42–78 y, - All healthy		BW, FM, "waist", "hip"			Low^a^	High
		*n* = 324, 4 RCTs	Whey-based beverage (whey blend, whey protein isolate or whey protein concentrate providing 12–55 g protein) vs. maltodextrin, glucose or casein		Intervention "did not influence body composition"	NA		
		*n* = 99, 2 RCTs	Whey-based beverage ("whey and peptides" or whey protein isolate providing 20–54 g protein) and energy deficit diet vs. maltodextrin		- One study reported a greater reduction in FM following the intervention as compared with the CHO supplementation - In the second study, there was no difference between the intervention and control group	NA		
		*n* = 215, 4 RCTs	Whey-based beverage (whey blend, non-specific whey, whey protein concentrate providing 23–60 g protein) and resistance training vs. (whey blend + resistance training), Gatorade, (Gatorade + creatinine), (Gatorade + whey + creatinine), maltodextrin, (whey + resistance training) or (whey + multimodal exercise programme)		In conjunction with resistance training, there was no difference in body composition measures between the intervention and control group	NA		
Lonnie 2020 [19]	- SR without MA of RCTs - RCTs published before 03/20 - Study duration: 18 d – 8 wk	*n* = 152, 5 RCTs, - Both sexes, - Mean age: 21.3–49.7 y, - No restriction regarding health status	Extracted plant proteins (lupin protein isolate, pea protein isolate, rice protein isolate, fava bean concentrate) vs. animal protein (milk protein isolate, milk protein + 1.6 g/d arginine, whey protein, egg whites, whey protein isolate) 25–70 g protein/d	BW, FM, WC	- 1 study: slight increase in BW and FM in both groups, but no differences between interventions - 1 study: increase in WC in milk protein group vs. lupin group, but no changes in BW or FM - 4 studies: no differences between the interventions	NA	Low^b^	Low
Zhao 2020 [20]	- SR with MA of RCTs - RCTs published before 11/19 - Study duration: 4–24 wk	*n* = 464, 10 RCTs, - Both sexes, - Mean age: 26–59.3 y, **-** All with hypercholesterolaemia	Plant protein (soya protein, barley protein, lupin protein or plant protein containing isoflavones) vs. animal protein (milk protein, casein, animal protein, whey protein or meat protein)	BW	Pooled MD (95% CI), fixed effect: − 0.41 kg (− 2.14, 1.33) *P* = 0.65	*I*^2^ = 0% *P* = 0.93 Chi^2^ = 3.70	Moderate	High
Badely 2019 [21]	- SR with MA of RCTs - RCTs published between 01.01.2000–30.05.2019 - Study duration: 4–32 wk	*n* = 1199, 19 RCTs, - Both sexes, - Mean age: 29.0–77.6 y, - Mean BMI: 25.0–36.5 kg/m^2^, - All with overweight or obesity	Whey protein (isolate, concentrate, extract, supplement, powder or hydrolysate)	WC	Pooled MD (95% CI), random effect: − 2.76 cm (− 3.83, − 1.69) *P* < 0.001	*I*^2^ = 100% *P* < 0.01	Low	Low
Donaldson 2019 [22]	- SR with MA of RCTs - RCTs published until 02/18 - Study duration: ﻿1–9 mo	*n* = 886, 13 RCTs, - Both sexes, - Mean age: 78.7–89.6 y, - Mean BMI: 18.4–27.3 kg/m^2^, - Residents of care homes, healthy, or with dementia, cognitive impairment or chronic illnesses	High-protein, nonmeat supplementation vs. control	BW	Pooled MD (95% CI), fixed effect: 1.11 kg (0.97, 1.24) *P* < 0.00001	*I*^2^ = 75% Chi^2^ = 52.86 df = 13 *P* < 0.01	Low	Low
Hsu 2019 [23]	- SR with MA of RCTs - RCTs published until 04/19 - Study duration: 12–16 wk	- Both sexes, - Age: 55.0–81.1 y, - All with sarcopenic obesity			(Pooled) MD (95% CI), fixed effect:			Low
		*n* = 26, 1 RCT (only men)	Protein shake (12 g protein) + resistance exercise vs. resistance exercise	BW	0.10 kg (− 9.23, 9.43) *P* = 0.98	NA	Low	
		*n* = 122, 2 RCTs (only women)	High-protein (1.2–1.4 g/ kg BW/d) vs. normal-protein (0.8–1.0 g/ kg BW/d), both low-calorie	FM	− 0.82 kg (− 1.34, − 0.30) *P* = 0.002	*I*^2^ = 58% Chi^2^ = 2.38 df = 1 *P* = 0.12	Low	
		*n* = 122, 3 RCTs	Protein supplementation + exercise vs. exercise	FM	0.57 kg (− 1.27, 2.41) *P* = 0.54	*I*^2^ = 0% Chi^2=^ 0.40 df = 2 *P* = 0.82	Low	
Li 2019 [24]	- SR with MA of RCTs - RCTs published until 07/18 - Study duration: 8–36 wk	*n* = 562, 15 RCTs, - Both sexes, - Mean age: 20.5–77.7 y, - Mean BMI: 24.0–28.2 kg/m^2^, - Mostly healthy, but also with HIV, limited mobility or hyperlipidaemia	Whey protein + resistance training vs. placebo + resistance training	FM	Pooled MD (95% CI), random effect: − 0.21 kg (− 0.69, 0.27) *P* = 0.39	*I*^2^ = 55% tau^2^ = 0.40 Chi^2^ = 33.17 df = 15 *P* = 0.004	Moderate	Moderate
Saboori 2019 [45]	- SR with MA of RCTs - RCTs published until 11/17 - Study duration: 4–39 wk	- Both sexes, - Mean age: 20.44–61.7 y, - Mean BMI: 21.8–27.6 kg/m^2^, - Healthy**,** exercising	Soya protein vs. control		Pooled MD (95% CI), fixed effect:			Moderate
		*n* = 203, 6 RCTs		BW	0.94 kg (− 2.41, 4.30) *P* = 0.58	*I*^2^ = 0% *P* = 0.99	Low	
		*n* = 160, 4 RCTs		FM	0.43 kg (− 2.18, 3.03) *P* = 0.74	*I*^2^ = 0% *P* = 0.54	Low	
Valenzula 2019 [25]	- SR with MA of RCTs - RCTs published until 05/19 - Study duration: 8wk–4mo	- Both sexes, - Age: 18–90 y, - Healthy - Mostly athletic active	Beef protein (isolated powder or lean beef providing 16.4–46 g protein/d) (1.3–2.2 g total protein/kg BW/d) vs. whey protein (1.7–2.2 g total protein/kg BW/d) or no protein supplementation (1.1–2.0 g total protein/kg BW/d)	FM	Pooled SMD (95% CI), random effect:			High
		*n* = 70, 4 RCTs	Beef protein vs. whey protein		0.07 (− 0.40, 0.54) *P* = 0.76	*I*^2^ = 0% Q = 0.012	Low	
		*n* = 224, 7 RCTs	Beef protein vs. no protein supplementation		0.15 (− 0.11, 0.42) *P* = 0.26	*I*^2^ = 0% Q = 1.238	Low	
van Baak 2019 [26]	- SR with MA of RCTs - Literature search period: NP - Study duration: 12 wk–12mo	*n* = 1168, 8 RCTs, - Both sexes, - Age: 18–75 y, - Generally healthy or with overweight or obesity	Protein (dietary advice to increase protein or supplementation) vs. control	BW after previous weight loss	Pooled SMD (95% CI), random effect: − 0.17 (− 0.29, − 0.05) *P* = 0.01	*I*^2^ = 0% tau^2^ = 0.00 Chi^2^ = 3.08 df = 11 *P* = 0.99	Moderate	Low
Bergia 2018 [27]	- SR with MA of RCTs - RCTs published between 2008–08/17 - Study duration: 6–52 wk	*n* = 572, 12 RCTs, - Only women, - Age: ≥ 19 y, - Healthy	Whey protein (6–48 g/d)		Pooled MD (95% CI), random effect:			Low
				BW	− 0.12 kg (− 0.90, 0.65) *P* NP	NP	Very low	
				FM	− 0.20 kg (− 0.67, 0.27) *P* NP	*I*^2^ = 80% tau^2^ = 0.452 Chi^2^ = 64.17 df = 13 *P* = 0.01	Low	
Dewansingh 2018 [28]	- SR with MA of RCTs - RCTs published until 03/16 - Study duration: 3–6 mo	*n* = 330, 5 RCTs, - Both sexes, - Mean age: 72–83.5 y, - Healthy or with prefrailty, frailty or mobility-limitation	Protein (8.6–40 g/d) vs. placebo	BW	Pooled MD (95% CI), fixed effect: 2.16 kg (0.93, 3.36) *P* = 0.0006	*I*^2^ = 0% Chi^2^ = 1.19 df = 4 *P* = 0.88	Low	Moderate
Hidayat 2018 [41]	- SR with MA of RCTs - RCTs published until 09/16 - Study duration: 12–72 wk	*n* = 542, 7 RCTs, - Both sexes, - Mean age: 60–80.8 y, - Healthy or with frailty, mobility-limitation, obesity or sarcopenia	Milk protein supplementation (20–40 g/d of whey protein, milk protein concentrate, casein or egg albumin) + resistance training		Pooled MD (95% CI), random effect:			High
				BW	1.02 kg (− 0.01, 2.04) *P* NP	*I*^2^ = 35% *P* = 0.15	Low	
				FM (kg)	0.30 kg (− 0.25, 0.86) *P* NP	*I*^2^ = 0% *P* = 0.43	Low	
Liao 2018 [29]	- SR with MA of RCTs - RCTs published between 1994–2017 - Study duration: less than 3–9 mo	- Both sexes, - Mean age: 76–87 y, - Mean BMI: 23.4–29.5 kg/m^2^, - With frailty or at risk for frailty	Protein supplementation (milk protein or leucine) + exercise vs﻿. placebo + exercise		Pooled SMD (95% CI), fixed effect:			Low
		*n* = 228, 4 RCTs		BW	0.32 (0.14, 0.50) *P* = 0.0005	*I*^2^ = 26%	Low	
		*n* = 90, 2 RCTs		FM	0.08 (− 0.33, 0.50) *P* = 0.69	*I*^2^ = 0% Chi^2^ = 0.02 df = 1 *P* = 0.89	Low	
Wirunsawanya 2018 [42]	- SR with MA of RCTs - Literature search period: NP - Study duration: 2 wk -15 mo	- Both sexes, - Mean age: 24.2–63 y, - All with overweight or obesity	Whey protein (20–75 g/d of isolate, concentrate or hydrolysate) vs. placebo or control Most control groups consumed a similar amount of protein to the whey protein group		Pooled MD (95% CI), random effect:			Moderate
		*n* = 335, 6 RCTs		BW	0.56 kg (0.31, 0.81) *P* NP	*I*^2^ = 0% *P* = 0.64	Moderate	
		*n* = 221, 3 RCTs		FM (kg)	1.12 kg (0.77, 1.47) *P* NP	*I*^2^ = 31% *P* = 0.23	Moderate	
		n = 134, 3 RCTs		FM (%)	− 0.34% (− 1.15, 0.46) *P* NP	*I*^2^ = 0% *P* = 0.74	Moderate	
		*n* = 311, 5 RCTs		WC	0.46 cm (− 0.66, 1.57) *P* NP	*I*^2^ = 84% *P* = 1.27	Moderate	
Chalvon-Demersay 2017 [43]	- SR without MA of observation studies and RCTs - Primary studies published until 03/16 - Study duration (observation studies): 5–7 y (NP for 1 study) - Study duration (RCTs): 2 wk – 4 y	- Both sexes, - Mean age: 18.2–68.4 y, - General population, healthy or with overweight, obesity, postmenopause, perimenopause, hypercholesterolaemia, diabetes or MS	Animal vs. plant protein (13–154 g protein/d)					Moderate
		*n* = 468,048, 4 observation studies (1 cross-sectional, 3 longitudinal)		BW (observation studies)	- 1 out of 2 studies: no effects of plant protein intake, but increase with animal protein intake - 1 out of 2 studies: decrease with plant protein intake, effect of animal protein intake not specified	NA	Low	
				WC (observation studies)	- 1 out of 2 studies: no effects of plant or animal protein intake - 1 out of 2 studies: decrease with plant protein intake, but increase with animal protein intake	NA	Moderate	
		*n* = 1807, 32 RCTs		BW (RCTs)	51/53 analyses did not report different effects of plant and animal protein		Moderate	
		*n* = 1017, 15 RCTs		FM (RCTs)	26/31 analyses did not report different effects of plant and animal protein		Moderate	
		*n* = 1171, 15 RCTs		WC (RCTs)	22/24 analyses did not report different effects of plant and animal protein		Moderate	
Liao 2017 [36]	- SR with MA of RCTs - RCTs published between 01/50–05/16 - Study duration: 8–24 wk	- Both sexes, - Mean age: 60.9–79.0 y, - Mean BMI: 25.0–33.3 kg/m^2^	Protein (3–40 g/d of protein, whey protein, casein, leucine metabolite, beta-hydroxy-betamethylbutyrate, leucine, essential amino acids or amino acid collagen peptide) + resistance exercise vs. control + resistance exercise		Pooled SMD (95% CI), random effect:			Low
		*n* = 633, 11 RCTs		FM (kg)	− 0.61 (− 0.93, − 0.29) *P* = 0.0002	*I*^2^ = 72% tau^2^ = 0.20 Chi^2^ = 35.34 df = 10 *P* = 0.0001	Moderate	
		*n* = 752, 15 RCTs		FM (%)	− 1.14 (− 1.67, − 0.60) *P* < 0.0001	*I*^2^ = 90% tau^2^ = 1.05 Chi^2^ = 155.98 df = 15 *P* < 0.00001	Moderate	
Kim 2016 [37]	- SR with MA of weight-loss RCTs - RCTs published until 01/15 - Study duration: 8–104 wk	*n* = 1174, 20 RCTs, - Weight-loss intervention, - Both sexes, - Mean age: 50.0–65.2 y, - Mean BMI: 29.6–36.6 kg/m^2^, - Healthy or with hyperinsulinaemia, heart failure, T2D or renal disease	< 25 EN% protein (< 1.0 g/kg BW/d) vs. > 25 EN% protein (> 1.0 g/kg BW/d)		Pooled MD (95% CI), random/fixed effect:			Low
		MA with EN% protein data: *n* = 885, 15 RCTs		BW	Random effect: − 0.54 kg (− 1.30, 0.23) *P* NP	*I*^2^ = 55% tau^2^ = 1.363 Chi^2^ = 40.25 df = 18 *P* = 0.002	Low	
		MA with g/kg BW/d protein data: *n* = 487, 11 RCTs		BW	Fixed effect: − 0.06 kg (− 0.66, 0.53) *P* NP	*I*^2^ = 15% tau^2^ = 0.248 Chi^2^ = 16.40 df = 14 *P* = 0.29	Low	
		MA with EN% protein data: *n* = 875, 14 RCTs		FM (kg)	Fixed effect: − 0.57 kg (− 0.98, − 0.15) *P* NP	*I*^2^ = 0% tau^2^ = 0.000 Chi^2^ = 13.08 df = 17 *P* = 0.73	Low	
		MA with g/kg BW/d protein data: *n* = 487, 12 RCTs		FM (kg)	Fixed effect: − 0.53 kg (− 1.08, 0.03) *P* NP	*I*^2^ = 0% tau^2^ = 0.000 Chi^2^ = 9.95 df = 14 *P* = 0.77	Low	
Clifton 2014 [38]	- SR with MA of weight loss/maintenance RCTs - RCTs published before 08/13 - Study duration: 52–208 wk	- Both sexes, - Age: ≥ 18 y, - Healthy or with T2D or PCOS	High-protein, low-CHO weight loss diet vs. control diet		Pooled SMD (95% CI), random effect:			Moderate
		*n* = 3492, 32 RCTs		BW	− 0.138 (− 0.231, − 0.046) *P* = 0.003	*I*^2^ = 36% Q = 48 *P* = 0.02	Low	
		*n* = 3202, 17 RCTs		FM	− 0.22 (− 0.32, − 0.12) *P* = 0.01	*I*^2^ = 0%	Low	
Johansson 2014 [44]	- SR with MA of weight-loss maintenance RCTs - RCTs published between 01/81–02/13 - Duration of weight loss maintenance period: 3–12 mo	*n* = 865, 6 RCTs, - Both sexes, - Mean age: 42–44 y, - Mean BMI: 29.3–39.0 kg/m^2^, - No information on health status	High-protein diet vs. control or placebo	BW after previous weight loss	Pooled MD (95% CI), random effect: − 1.5 kg (− 2.1, − 0.8) *P* < 0.001	*I*^2^ = 0% *P* = 0.70	Low	Low
Miller 2014 [40]	- SR with MA of RCTs - RCTs published until 11/12 - Study duration: 6–36 wk	- Both sexes, - Age: 18–66 y, - Generally healthy or with overweight, obesity or hyperlipidaemia	Whey protein (0.3–1.35 g/kg BW/d of isolate, concentrate or hydrolysate) vs. CHO		Pooled MD (95% CI), random effect:			Low
		*n* = 144, 3 RCTs	WPR (whey protein as replacement for other energy sources)	BW	− 1.85 kg (− 6.73, 3.02) *P* NP	*P* = 0.87	Very low	
		*n* = 259, 6 RCTs	WPS (whey protein as a supplement to the diet without dietary modification)	FM (kg)	− 0.21 kg (− 2.16, 1.75) *P* NP	*P* = 0.97	Very low	
		*n* = 118, 2 RCTs	WPR	FM (kg)	− 0.60 kg (− 4.08, 2.88) *P* NP	*P* = 0.46	Very low	
		*n* = 118, 2 RCTs	WPR	WC	− 0.92 cm (− 4.86, 3.03) *P* NP	*P* = 0.69	Very low	
Pedersen 2013 [33]	- SR without MA of RCTs and prospective cohort studies - Primary studies published between 01/00–12/11 - Study duration: NP	*n* = 136,729, 9 studies (2 RCTs, 7 prospective cohort studies) - Both sexes, - Generally healthy	RCTs: high-protein vs. low/normal-protein Cohort studies: protein intake	BW, FM & WC	*"The evidence is assessed as inconclusive regarding the relation of protein intake to change in BW, (…) WC, (…) body composition"*	NA	-Low for 7 analyses^c^ -Moderate for 1 analysis^d^	Moderate
Schwingshackl 2013 [32]	- SR with MA of RCTs - RCTs published before 08/12 - Study duration: 12–24 mo	- Both sexes, - Healthy or with T2D	High-protein (≥ 25 EN%) vs. low-protein (≤ 20 EN%), both low-fat (≤ 30 EN%)		Pooled MD (95% CI), random effect:			High
		*n* = 971, 13 RCTs		BW	− 0.39 kg (− 1.43, 0.65) *P* = 0.46	*I*^2^ = 0% tau^2^ = 0.00 Chi^2^ = 10.90 df = 12 *P* = 0.54	Low	
		*n* = 913, 10 RCTs		FM (kg)	− 0.59 kg (− 1.32, 0.13) *P* = 0.11	*I*^2^ = 0% tau^2^ = 0.00 Chi^2^ = 5.24 df = 9 *P* = 0.81	Low	
		*n* = 727, 8 RCTs		WC	− 0.98 cm (− 3.32, 1.37) *P* = 0.41	*I*^2^ = 72% tau^2^ = 7.86 Chi^2^ = 25.20 df = 7 *P* = 0.0007	Low	
Fogelholm 2012 [34]	- SR without MA of prospective cohort studies - Primary studies published from 2000 - Follow up: 5 y	*n* = 46,579, 2 prospective cohort studies, - Both sexes, - Age: 30–64 y, - BMI: 23.4–26.1 kg/m^2^	Total protein	BW & WC	*"The intake of total proteins did not show consistent associations with weight gain"* *"The role of protein in prevention of an increase in weight or WC was inconsistent: the two identified studies reported a neutral (…) or negative (…) association"* *"The results suggested that the proportion of macronutrients in the diet was not important in predicting changes in weight or WC"*	NA	-Moderate: WC -Low: BW	Moderate
	- SR without MA of RCTs - RCTs published from 2000 - Study duration: unclear	*n* = 1178, 4 RCTs, - Both sexes, - Age: 18–75 y, - BMI: 27–45 kg/m^2^, - One study with T2D and another also included participants with other comorbidities	Total protein	BW after previous weight loss	*"A high-protein, low carbohydrate diet protected against weight regain in on study, but no effects were observed in three other studies."* *- "The results on the role of dietary macronutrient composition in prevention of weight regain (after prior weight loss) were inconclusive"*	NA	-Moderate: BW, WC -Low: FM	
Santesso 2012 [35]	- SR with MA of RCTs - RCTs published before 08/11 - Study duration: 28–365 d	- Both sexes, - Mean age: 26–62 y, **-** Healthy or with overweight, obesity, diabetes, hyperlipidaemia, hypertriglyceridaemia, cardiac risk factors, MS or PCOS	High-protein (27 EN%) vs. low-protein (18 EN%) diet		Pooled SMD (95% CI), random effect:			High
		MA with change values: *n* = 2326, 38 RCTs		BW	− 0.36 (− 0.56, − 0.17) *P* = 0.0002	*I*^2^ = 77% tau^2^ = 0.25 Chi^2^ = 167.76 *P* < 0.00001	Low	
		MA with end values: *n* = 1945, 38 RCTs		BW	− 0.07 (− 0.16, 0.02) *P* = 0.13	*I*^2^ = 0% tau^2^ = 0.00 Chi^2^ = 27.83 *P* = 0.94	Low	
		MA with change values: *n* = 1214, 15 RCTs		WC	− 0.43 (− 0.69, − 0.16) *P* = 0.00001	*I*^2^ = 75% tau^2^ = 0.19 Chi^2^ = 60.83 *P* < 0.00001	Low	
		MA with end values: *n* = 699, 9 RCTs		WC	− 0.05 (− 0.24, 0.13) *P* = 0.57	*I*^2^ = 26% tau^2^ = 0.02 Chi^2^ = 12.21 *P* = 0.20	Low	
Wycherley 2012 [39]	- SR with MA of weight loss RCTs - RCTs published before 05/11 - Study duration: ≥ 4 wk	- Both sexes, - Age ≥ 18 y, - Healthy or with overweight, obesity, prediabetes, T2D, hyperinsulinaemia, PCOS, heart failure or MS	High-protein vs. standard-protein diet, both energy-restricted & low-fat					Low
		*n* = 1010, 22 RCTs		BW	Pooled MD (95% CI), random effect: − 0.79 kg (− 1.50, − 0.08) *P* = 0.03	*I*^2^ = 71% Chi^2^ = 75.64 df = 22 *P* < 0.00001	Low	
		*n* = 765, 17 RCTs		FM (kg)	Pooled MD (95% CI), fixed effect: − 0.87 kg (− 1.26, − 0.48) *P* < 0.0001	*I*^2^ = 1% Chi^2^ = 17.13 df = 17 *P* = 0.45	Low	
Lepe 2011 [31]	- SR without MA of RCTs - Literature search period: NP - Study duration: 6–24 mo	*n* = 645, 8 RCTs, - Both sexes, - Age: 18–70 y, - BMI: 25–43 kg/m^2^, - Healthy or with diabetes or hyperinsulinaemia	High-protein diet (25–40 EN% protein) vs. conventional or high-fat or high-CHO diet (12–24 EN% protein)	BW	*"The average weight loss of the eight studies in the high-protein diet was 6.3 kg and in the standard diet was 5.0 kg. Although half of the studies showed a higher weight loss with a high-protein diet, three out of four studies with the longest intervention show no statistical difference in weight loss. In this systematic review it was observed that the long-term effect of high-protein diets is neither consistent nor conclusive"* *"Additionally, the non-statistical difference observed in the majority of the studies conducted for more than 12 months, suggested a diminished trend of weight loss with the lenght of intervention"*	NA	Low	Low
Summerbell 2009 [30]	- SR without MA of prospective cohort studies - Primary studies published until 12/07 - follow up: > 1 y	Total protein: *n* = 81,286, 8 cohort studies, Plant protein: none cohort studies, Animal protein: *n* = 42,696, 1 cohort study, - Both sexes, - Age NR in all studies, - No information on health status	Total protein, plant protein, animal protein	BW & WC	**Overall***: "SUMMARY STATEMENT of the association between proteins* *and subsequent excess weight gain and obesity: The substantial evidence reviewed suggests that levels of protein intake, regardless of source, are not associated with subsequent* *excess weight gain or obesity, although the results were inconsistent."* **plant protein***: "exposure with no studies found"* **animal protein***: "no clear associations were observed between animal protein intake and subsequent changes in waist circumference across both male and female subgroups"*	NA	-Low: BW & total protein -Very low: WC & total protein -Low: WC & animal protein	Low

*AMSTAR 2* A Measurement Tool to Assess Systematic Reviews 2, *BMI* body mass index, *BW* body weight, *CHO* carbohydrates, *CI* confidence interval, *d* day(s), *df* degrees of freedom, *EN%* percentage of energy intake, *FM* fat mass, *MA* meta-analysis/meta-analyses, *mo* month(s), *MS* metabolic syndrome, *NA* not applicable, *NP* not provided, *NS* not statistically significant, *PCOS* polycystic ovary syndrome, *Q* Cochran’s Q, *RCT(s)* randomised controlled trial(s), *sig* statistically significant, *SMD* standardised mean difference, *SR(s)* systematic review(s), *T2D* type 2 diabetes mellitus, *wk* week(s), *MD* weighted mean difference, *WC* waist circumference, *WPR* whey protein as replacement for other energy sources, *WPS* whey protein as a supplement to the diet without dietary modification, *y* year(s)

^a^A total of 3 NutriGrade assessments were conducted, all received a rating of low

^b^A total of 3 NutriGrade assessments were conducted, all received a rating of low

^c^Cohort studies evidence on BW & total protein, BW & plant protein, WC & total protein, WC & animal protein, WC & plant protein and RCT evidence on BW & total protein and body composition & total protein

^d^Cohort studies evidence on BW & animal protein

### Methodological quality

Overall scores of AMSTAR 2 for each included SR are summarised in Table 2. Supplementary Material 6 provides a more detailed overview showing the assessments of each individual item. Methodological quality of the included SRs as assessed with AMSTAR 2 was high for eight SRs, moderate for ten SRs, and low for 15 SRs. One SR rated as “critically low” by AMSTAR 2 was excluded from the current work (Fig. 1).

### Associations/effects of protein intake and outcome-specific certainty of the evidence

The impact of total protein intake on BW-related outcomes was investigated in 29 RCTs [18–29, 31, 32, 35–42, 44–50]; study characteristics are shown in Table 2. Four SRs included cohort studies [30, 33, 34, 43]; the results are shown in Table 2. Out of the 98 NutriGrade ratings of outcome-specific certainty of evidence, twelve were rated very low, 61 low and 25 moderate, respectively; none was ranked as high. Overall scores of NutriGrade for each SR are summarised in Table 2. Supplementary Material 7 provides a more detailed account showing the assessments of each individual NutriGrade item.

(1) Effects of the amount of protein intake on BW, FM and WC in studies without energy restrictions

The effect of the amount of protein intake on BW, FM and/or WC in adults under predominantly isoenergetic conditions was investigated in 16 SRs, among them 13 SRs with MA [21, 24, 25, 27, 32, 35, 40, 42, 45–49] and 3 SRs without MA [18, 30, 33] (Table 2). SRs with MA defined different inclusion criteria on protein intake: ≥ 25 EN% vs. ≤ 20 EN% [32], differences of ≥ 3 EN% [49] and ≥ 5 EN% [35] between both treatments, respectively, or supplemental protein intake of 20–50 g/day [42], 0.3–1.35 g/kg BW/day [40] and 6–48 g/day [27]. Five SRs with MA did not consider the amount of protein as inclusion criteria [24, 45–48]. In seven out of 13 SRs with MA, the duration of protein intervention was defined as inclusion criteria with ≥ 2 weeks [21, 42], ≥ 4 weeks [25, 40, 49], ≥ 6 weeks [24] and ≥ 12 months [32]. Intervention was conducted by supplementation with whey protein [18, 21, 24, 27, 40, 42, 46], soya protein [45, 46, 48], beef protein [25] or dietary proteins without further specification [32, 33, 35, 47, 49].

Four out of 10 SRs with MA of RCTs showed a reduction of BW [35, 42, 47, 49], whereas six did not find any effects [27, 32, 40, 45, 46, 48] (Table 2). The impact on FM was investigated in nine SRs with MA. Again, most of them did not find an effect of the quantity of protein consumed on FM [24, 25, 27, 32, 40, 45, 46]. However, Vogtschmidt et al. [49] and Wirunsawanya et al. [42] found a decrease in FM after higher protein consumption (Table 2). Seven SRs with MA considered WC; five of them found no effects [32, 40, 42, 48, 49], whereas two found a decrease in WC by protein intervention [21, 35]. A multivariable meta-regression analysis did not show a dose–response relationship between the additional protein intake by intervention and the changes in BW as well as WC taking into account differences in the intake of protein (EN%), total energy and in CHO (EN%) between intervention and control groups [35].

To sum up, most SRs with MA did not find an effect of the amount of protein on BW, FM and WC in studies without energy restriction. For BW, FM and WC, the majority of the included SRs with and without MA reached at least a low outcome-specific certainty of evidence, and the majority of all included SRs reached at least a methodological quality of moderate (Table 2). Therefore, the overall certainty of evidence was graded as “possible” that the amount of protein intake does not affect BW, FM and WC in adults under isoenergetic conditions.

(2) Effects of the amount of protein intake on BW, FM and WC in studies without energy restrictions in the subgroup of older adults

The impact of the amount of protein intake on BW under predominantly isoenergetic study conditions in older adults was investigated in five SRs with MA [22, 23, 28, 29, 41]; four considered FM [23, 29, 36, 41] (Table 2). WC was not investigated by any SR. Only Donaldson et al. [22] defined protein intake as inclusion criterion, which had to be at least 10 g/day higher in the protein intervention group compared to the control group. The period of intervention was not an inclusion criterion in any SR except Hidayat et al. [41], which included only RCTs with an intervention duration of more than twelve weeks. Intervention was performed for 3 to 6 months [28], 12 to 72 weeks [41], 8 to 24 weeks [36] and up to 9 months [22, 29]. Intervention was conducted by supplementation of milk protein [28, 41] or by intake of non-meat protein and mixture of (dietary) protein types except meat [29, 36] (Table 2). Two SRs with MA did not provide any details on the type of protein [22, 23].

Five SRs with MA investigated the effect of the amount of protein intake on BW; three of them found an increase in BW in older adults [22, 28, 29], whereas two did not detect an effect [23, 41] (Table 2). The impact on FM was investigated in four SRs with MA. Three of them did not find an effect on FM [23, 29, 41]. Only Liao et al. [36] found a decrease in FM (Table 2).

To sum up, most SRs with MA in older adults found an increase in BW due to a higher protein intake without modulating FM. However, the majority of the included SRs reached only a low outcome-specific certainty of evidence and had only low methodological quality (Table 2). Consequently, the overall certainty of evidence was graded as “insufficient” that the amount of protein ingested may increase BW without affecting FM in older adults under isoenergetic conditions. Since no SR was identified for WC, evidence was also “insufficient”.

(3) Effects of the amount of protein intake on BW, FM and WC in studies with energy restriction

The effect of the amount of protein intake on BW under hypoenergetic study conditions was investigated in six SRs with MA [27, 35, 37–39, 50] and in one SR without MA [31]; the effect on FM was studied in six SRs with MA [23, 27, 37–39, 50] (Table 2). Two SRs with MA investigated WC [35, 50]. Protein intervention and study duration differed strongly between SRs (Table 2). For example, in the SR with MA of Wycherley et al. [39] protein intake differed at least by 10 EN% between energy-restricted diet with high vs. standard-protein content with an intervention of ≥ 4 weeks. In the SR with MA of Kim et al. [37], protein intake was > 25 EN% (or > 1.0 g/kg BW/day) vs. < 25 EN% (or < 1.0 g/kg BW/day) for at least 8 weeks (Table 2). Intervention was conducted with a mixture of (dietary) protein types as part of a whole diet approach (Table 2).

Four out of six SRs with MA found a decrease in BW in response to a higher protein intake under hypoenergetic conditions [35, 38, 39, 50], whereas two did not observe an effect of the protein intervention [27, 37]. FM was investigated by six SRs with MA as outcome [23, 27, 37–39, 50], and five of them found a decrease in FM [23, 37–39, 50]. WC decreased in the MA of Santesso et al. [35], but did not change in the MA of Zhang et al. [50] (Table 2).

To sum up, the majority of SRs with MA found a stronger decrease in BW by a higher intake of protein under hypoenergetic conditions, but most of the included SRs reached a low outcome-specific certainty of evidence and a low methodological quality. Therefore, the overall certainty of evidence for an impact of protein intake on BW under hypoenergetic conditions was rated as “insufficient”. In most SRs with MA, FM decreased in response to a higher intake of protein under energy restriction, but the majority of included SRs reached only a low outcome-specific certainty of evidence and a low methodological quality. Hence, the overall certainty of evidence that a protein-rich hypoenergetic diet affects FM is “insufficient”. For WC, the effect of protein intake was also judged to be “insufficient” as consistent risk associations/effects were lacking (Table 2).

(4) Effects of the amount of protein intake on maintenance of BW, FM and WC after BW reduction in subjects with overweight/obesity

Effects of the amount of protein intake on the maintenance of BW after a previous weight loss in adults with overweight or obesity through an energy-restricted diet were investigated in three SRs, two of them with MA [26, 44] and one without MA [34]. We did not identify any SRs with FM as outcome parameter. One SR without MA investigated the effects on WC [34]. As shown in Table 2, all SRs considered studies with highly different protein intakes (e.g., addition of 30–48 g/day or addition of 10–15 EN%) with a duration of 3 to 12 months [26], 3 months to 3 years [44] or ≥ 6 months [34].

Both SRs with MA found a further decrease in BW with higher protein intake compared to lower intake after initial weight loss [26, 44]. In the SR without MA, total protein intake did not show consistent associations with changes in BW or WC [34] (Table 2).

To sum up, a further decrease in BW after BW reduction was found in both SRs with MA, which may contribute to long-term weight maintenance. Yet, as most SRs reached a very low outcome-specific certainty of evidence and a low methodological quality, the overall certainty of evidence was graded as “insufficient” that a higher protein intake prevents a regain of BW in adults with overweight or obesity after achieving weight loss by means of an energy-restricted diet. Due to the lack of SRs on FM, the overall certainty of evidence for an association between the amount of protein intake and FM was “insufficient”. For WC, only a single SR without MA is available showing different associations with protein intake on the basis of two cohort studies. Therefore, the overall certainty of evidence was judged as “insufficient” without considering the grading criteria.

(5) Effects of the type of protein on BW, FM and WC

The effects of the type of protein on BW were investigated in four SRs. Two of them included an MA and were based on RCTs [20, 40]. Two SRs without MA included intervention studies [19, 43], Chalvon-Demersay et al. [43] additionally included cohort studies. FM was investigated in two SRs with MA [25, 40] and two SRs without MA [19, 43]. WC was investigated in one SR with MA [40] and in two SRs without MA [19, 43]. The participants were mostly healthy [25, 40, 43], partly with metabolic impairment [43], or they suffered from hypercholesterolaemia [20]. Two SRs with MA compared the effect of plant vs. animal protein for 4 to 24 weeks [20] and 5 to 7 years [43], respectively. Another SR with MA studied the effect of whey protein vs. other proteins [40]. SRs without MA compared a variety of plant proteins (mostly soya) with protein of animal origin (mostly casein) [43] or alternative plant proteins (e.g., lupine, pea, fava bean, rice, oat, hemp, lentil) with milk protein [19] (Table 2).

Neither of the SRs with MA found an effect of the type of protein on BW [20, 40]; the results of SRs without MA are consistent since most of the included RCTs did not report different effects of animal and plant protein on BW [19, 43]. Valenzuela et al. [25] compared the intake of beef protein and whey protein on FM in healthy adults, mostly athletes, but did not find differences in these types of protein. Miller et al. [40] examined whey protein vs. other proteins, but did not find a specific effect of whey protein. Supplementation of whey protein did not affect WC compared to other proteins [40]. Most RCTs in SRs without MA did not find an effect of the protein type on WC [19, 43]. Results from cohort studies are unclear [43] (Table 2).

To sum up, neither of the SRs with MA showed an effect of protein type on BW, FM and WC. The results from SRs without MA on these parameters remain unclear. As the majority of included SRs reached a low methodological quality and a low outcome-specific certainty of evidence for each outcome, the overall certainty of evidence that the type of protein may influence BW, FM and WC was considered to be “insufficient”.

## Discussion

The aim of this umbrella review was to assess whether the amount and type of protein may affect BW, FM and WC in adults with consideration of the overall certainty of evidence. To our knowledge, this umbrella review is the first to provide a summary evidence assessment of previous SRs. Our major finding is that there is “possible” evidence that under isoenergetic study conditions the amount of protein did not affect BW, FM and WC in the general adult population. For further settings, such as (i) older adults, (ii) hypoenergetic diets, and (iii) diets following weight reduction, the overall certainty of evidence was graded as “insufficient”. Moreover, the evidence for an influence of the type of protein on BW, FM and WC was also “insufficient”.

Our finding that the amount of protein did not affect BW and related parameters in studies without intended energy restriction may be explained by the combination of several factors, such as methodological limitations (e.g., ad libitum food consumption in free-living subjects, which is determined by a couple of exogeneous factors; high variation in the duration of interventions). Furthermore, the assumed physiological effect of a high protein intake (e.g., thermogenic effect) seems to be negligible in settings without energy restriction.

For older adults, albeit with “insufficient” evidence, most of the considered SRs with MA found an increase in BW in response to higher protein intakes (under isoenergetic conditions), which could not be explained by an increase in FM. This suggests that older people may benefit from a high-protein diet since the observed increase in BW may be partly explained by fat-free mass. Some nutrition societies (e.g., German Nutrition Society, ESPEN) recommend a higher protein intake (e.g., 1.0 vs. 0.8 g/kg BW/day) for older adults to combat age-related losses of muscle mass and muscle strength [51, 52]. In five out of six SRs, the higher protein intake was achieved via administration of milk protein including whey protein [22, 28, 29, 36, 41] (Table 2), which was mostly combined with physical activity [22, 23, 29, 36, 41]. Whey protein has been discussed to be an optimal protein source to support muscle protein synthesis at rest and following resistance training to induce muscle hypertrophy and strength gains. The anabolic effect of whey protein is explained by its amino acid content (high EAA, branched-chain amino acids, particularly leucine), rapid digestibility, and high availability within the plasma and muscle tissue upon consumption to induce muscle protein synthesis [8].

Under hypoenergetic study conditions, most of the SRs with MA showed that a higher protein intake lowers BW and FM more than a lower protein intake under free-living conditions. The overall certainty of evidence was only rated as “insufficient” as the demands on methodological quality were not fulfilled. Protein intake with a high-protein diet was > 1.0 g/kg BW/day in two SRs of RCTs [23, 37]. Effects attributed to protein, such as increased diet-induced thermogenesis and hunger suppression/satiety, seem to be especially relevant under the setting of energy restriction and weight reduction [53, 54].

Whether the origin of protein, e.g., from animal vs. plants, has an impact on BW, FM and WC remains unclear due to “insufficient” evidence. Current dietary guidelines recommend plant-based diets. In addition to vegetables, fruit and cereals, this also implies a regular consumption of protein sources of plant origin, such as legumes and nuts [55]. Due to the great importance of plant-based foods as part of a healthy and sustainable diet, further studies on the role of the protein type on BW, FM and WC are needed.

Currently, a lot of SRs are available on the impact of protein quantity on BW and associated parameters in different settings. This allows differentiated statements on the impact of protein amount on BW, FM and WC. Considering the type of protein, however, only a few SRs could be found; these investigated quite different questions (e.g., animal vs. plant protein; whey vs. other protein). Moreover, the investigation of the effects of protein type requires an isonitrogeneous diet, which is difficult to implement if natural foods are used. This is only practicable by using protein supplements or isolates/concentrates. If natural food is used as a protein source, nutrient composition and energy density of the diets may be different. This might affect hunger and satiety, thereby influencing energy balance and thus BW in the longer term. This point concerns studies on protein type, but also on the amount of protein. An increased intake of dietary protein requires a simultaneous decrease in either CHO or fat intake to ensure an equal supply of energy. This aspect of energy substitution is important, and it is rather questionable whether this was considered in all SRs included. This problem might have been resolved by stricter criteria for the selection of SRs, but this would have considerably reduced the number of relevant SRs. Our search and selection strategy can also be critically questioned with regard to the study collective. The aim of our umbrella review was to investigate the health-promoting effects of the protein. Some SR included both healthy adults and also subjects at risk of obesity-associated diseases (Table 2). A further limitation could be that the literature search was last updated in 01/2022, and thus, it cannot be ruled out that any very recently published SR regarding the effect of protein intake on health outcomes have not been included in the present umbrella review.

About one-fourth of all SRs of RCTs defined the additional protein intake by intervention or the difference in protein intake compared to control treatment as criteria for eligibility [22, 32, 35, 37, 39, 49], whereas most SRs did not [18–21, 23–29, 31, 33, 34, 36, 38, 40–48, 50]. The protein intake by intervention was mostly given in grammes per day [18, 19, 21, 22, 25, 27–29, 36, 41–46, 48] and partly in grammes per kg BW [21, 23, 36, 37, 39–41, 45, 50]. Sometimes, total protein intake for both treatments was provided in EN% [31–35, 37–39, 44, 47, 49]. In two SRs, data on protein intake were completely missing [20, 24]. These differences make it difficult to compare the different SRs with each other to derive a specific amount of protein associated with a preventive effect on BW.

We included the results of all relevant SRs, regardless of overlap as our purpose was to present and describe the current body of SR evidence. Having assessed the extent of primary study overlap between the SRs, bias due to multiple inclusion of the same primary studies in different SRs is unlikely, as the primary study overlap is only small at 2.2%.

In conclusion, it is rather unlikely that the amount of protein may affect BW, FM and WC in adults under isoenergetic conditions. The impact of a high-protein diet concerning body composition and the reduction of BW under hypoenergetic conditions remains unclear. In addition, the evidence for an influence of the type of protein on BW, FM and WC is “insufficient”. Thus, further SRs of RCTs with high methodological quality are mandatory. This also implies a sufficient number of well-controlled and well-designed RCTs.

### Supplementary Information

Below is the link to the electronic supplementary material.

Supplementary file1 (DOCX 16 KB)

Supplementary file2 (DOCX 32 KB)

Supplementary file3 (DOCX 63 KB)

Supplementary file4 (DOCX 37 KB)

Supplementary file5 (XLSX 42 KB)

Supplementary file6 (DOCX 35 KB)

Supplementary file7 (XLSX 42 KB)

Supplementary file8 (XLSX 14 KB)

## Abbreviations

AMSTAR 2: A Measurement Tool to Assess Systematic Reviews 2
BW: Body weight
CHO: Carbohydrates
EAA: Essential amino acids
EN%: Percentage of energy intake
FM: Fat mass
MA: Meta-analysis/meta-analyses
RCT(s): Randomised controlled trial(s)
SR(s): Systematic review(s)
WC: Waist circumference
