# Supplementary material for: Protein intake and body weight, fat mass and waist circumference: an umbrella review of systematic reviews for the evidence-based guideline on protein intake of the German Nutrition Society
Source: Eur J Nutr. 2023 Oct 4;63(1):3–32. doi: 10.1007/s00394-023-03220-x (PMC10799103; doi:10.1007/s00394-023-03220-x)
Supplement: Supplementary file 3 — Supplementary file3 (DOCX 63 KB) [file 394_2023_3220_MOESM3_ESM.docx]

**Supplementary Material 3.** NutriGrade scoring tool.

This supplement provides an overview of the applied NutriGrade scoring system. Detailed guidance and information on the allocation of points can be found here: Schwingshackl L, Knüppel S, Schwedhelm C, Hoffmann G, Missbach B, Stelmach-Mardas M, Dietrich S, Eichelmann F, Kontopanteils E, Iqbal K, Aleksandrova K, Lorkowski S, Leitzmann MF, Kroke A, Boeing H: Perspective: NutriGrade: A scoring system to assess and judge the meta-evidence of randomized controlled trials and cohort studies in nutrition research. Adv Nutr 2016;7:994–1004.

**NutriGrade scoring system for SRs with MA of RCTs**

1. Risk of bias/ study quality/ study limitations **(3 P)**
   1. No quantitative and descriptive information available (0 P)
   2. Risk of bias (3 P)
      1. Sequence generation^1^
      2. Allocation concealment^1^
      3. Blinding of participants and personnel^1^
      4. Blinding of outcome assessment personnel^1^
      5. Incomplete outcome^1^
      6. Selective reporting^1^
   3. Study quality (2 P)^2^
2. Precision **(1 P)**
   1. <400 participants OR 400-2000 participants, but 95% CI overlaps the null value (0 P)
   2. >2000 participants OR 400-2000 participants, but 95% CI excludes the null value (1 P)
3. Heterogeneity **(1 P)**
   1. ≤ 5 studies (0 P)
   2. 6-9 studies (if ≥10 studies; multiply points by 2):
      1. I^2^ (H^2^ and/or tau^2^) (0.1 P)
      2. CIs for I^2^ (0.1 P)
      3. If I^2^ <40% (0.3 P) skip **iv**
      4. Modelling detected heterogeneity (I^2^≥40%) with random effects model (0.1 P)
         1. Exploring detected heterogeneity with subgroup analysis or meta-regression (0.1 P)
         2. Sensitivity analyses with higher levels of heterogeneity (0.1 P)
4. Directness **(1 P)**
   1. Differences in population; differences in intervention; surrogate markers; network meta-analysis (0 P)
   2. No important differences in population or intervention; hard clinical outcome (1 P)
5. Publication bias **(1 P)**
   1. <5 studies OR evidence for severe bias with test or plot OR publication bias not assessed (0 P)
   2. No evidence for publication bias with test or plot (5-9 studies) OR evidence for moderate/small amount of publication bias with test or plot (0.5 P)
   3. No evidence for publication bias with test or plot (≥10 studies) (1 P)
6. Funding bias **(1 P)**
   1. Industry funding OR conflict of interest (0 P)
   2. Private institutions, foundations, non-governmental organizations (0.5 P)
   3. Academic institutions, research institutions (1 P)

**2**

1. Study design **(+ 2 P)**

**Overall score**^3^

P: point(s); RCT: randomized controlled trial.

^1^ ≥2/3 of studies low risk of bias = 0.5 P; >1/3 of studies high risk of bias OR not assessed = 0 P; unclear risk of bias = 0.25P)

^2^ ≥2/3 of overall score = 2 P; ≥1/3 of overall score = 1 P; otherwise = 0 P

^3^ 0-3.99: very low evidence; 4-5.99: low evidence; 6-7.99: moderate evidence; ≥8: high evidence

**NutriGrade scoring system for SRs with MA of cohort studies**

1. Risk of bias/ study quality/ study limitations **(2 P)**
   1. No information available (0 P)
   2. Risk of bias (2 P)
      1. Ascertainment of exposure^1^
      2. Adjusted basic & outcome relevant model^1^
      3. Assessment of outcome^1^
      4. Adequacy of follow-up duration^1^
   3. Study quality (2 P)^2^
2. Precision **(1 P)**
   1. <500 events OR ≥500 events but 95% CI overlaps the null, and includes important benefit (RR: <0.8) or harm (RR: >1.2) (0 P)
   2. ≥500 events and the 95% CI excludes the null values; ≥500 events but 95% CI overlaps the null, and excludes important benefit (RR: <0.8) or harm (RR: >1.2) (1 P)
3. Heterogeneity **(1 P)**
   1. ≤ 5 studies (0 P)
   2. 6-9 studies (if ≥10 studies; multiply by 2):
      1. I^2^ (H^2^ and/or tau^2^) (0.1 P)
      2. CIs for I^2^ (0.1 P)
      3. If I^2^ <40% (0.3 P) skip iv
      4. Modelling detected heterogeneity (I^2^ ≥40%) with random effects model (0.1 P)
         1. Exploring detected heterogeneity with subgroup analysis or meta-regression (0.1 P)
         2. Sensitivity analyses with higher levels of heterogeneity (0.1 P)
4. Directness **(1 P)**
   1. Differences in population; differences in intervention; surrogate markers; network meta-analysis (0 P)
   2. No important differences in population or intervention; hard clinical outcome (1 P)
5. Publication bias **(1 P)**
   1. <5 studies OR evidence for severe bias with test or plot OR publication bias not assessed (0 P)
   2. No evidence for publication bias with test or plot (5-9 studies) OR evidence for moderate/small amount of publication bias with test or plot (0.5 P)
   3. No evidence for publication bias with test or plot (≥10 studies) (1 P)
6. Funding bias **(1 P)**
   1. Industry funding OR conflict of interest (0 P)
   2. Private institutions, foundations, non-governmental organizations (0.5 P)
   3. Academic institutions, research institutions (1 P)
7. Effect size **(2 P)**
   1. No effect (HR/RR: 0.80-1.20) (0 P)
   2. Moderate effect size (HR/RR: <0.80-0.50 or >1.2-2.00) (1 P)
   3. Large effect size (HR/RR: <0.50 or >2.00) (2 P)
8. Dose-response **(1 P)**
   1. No dose-response relationship (corresponding statistical test non- significant) (0 P)
   2. Linear and/ or non-linear dose-response relationship (corresponding statistical test significant) (1 P)

**Overall score**^3^

P: point(s); RR: risk ratio.

^1^ ≥2/3 of studies low risk of bias = 0.5 P; >1/3 of studies high risk of bias OR not assessed = 0 P; unclear risk of bias = 0.25 P)

^2^ cut-off for different quality scale (≥3/4 of overall score= 2 P; ≥1/2 of overall score= 1 P; <1/2 of overall score= 0 P); i.e. **Newcastle-Ottawa Scale** (mean): ≥7= 2 P; 4-6.9= 1 P; 0-3.9= 0 P;

^3^ 0-3.99: very low evidence; 4-5.99: low evidence; 6-7.99: moderate evidence; ≥8: high evidence

**NutriGrade scoring system for SRs without MA of RCTs**

1. Risk of bias/ study quality/ study limitations **(3 P)**
   1. No quantitative and descriptive information available (0 P)
   2. Risk of bias (3 P)
      1. Sequence generation^1^
      2. Allocation concealment^1^
      3. Blinding of participants and personnel^1^
      4. Blinding of outcome assessment personnel^1^
      5. Incomplete outcome^1^
      6. Selective reporting^1^
   3. Study quality (2 P)^2^
2. Precision **(1 P)**
   1. <400 participants (0 P)
   2. ≥400 participants (1 P)
3. Heterogeneity **(1 P)**
   1. >1/3 of included studies have an inconsistent result (i.e. point estimates and/or 95% CI did not overlap between studies) (0 P)
   2. ≥2/3 of included studies have a consistent result (i.e. point estimates and/or 95% CI did overlap between studies) (1 P)
4. Directness **(1 P)**
   1. Differences in population; differences in intervention; surrogate markers; network meta-analysis (0 P)
   2. No important differences in population or intervention; hard clinical outcome (1 P)
5. Funding bias **(1 P)**
   1. Industry funding OR conflict of interest (0 P)
   2. Private institutions, foundations, non-governmental organizations (0.5 P)
   3. Academic institutions, research institutions (1 P)

**2**

1. Study design **(+ 2 P)**

**Overall score**^3^

95% CI: confidence intervals; P: point(s); RCT: randomized controlled trial.

^1^ ≥2/3 of studies low risk of bias = 0.5 P; >1/3 of studies high risk of bias OR not assessed = 0 P; unclear risk of bias = 0.25P)

^2^ ≥2/3 of overall score = 2 P; ≥1/3 of overall score = 1 P; otherwise = 0 P

^3^ 0-3.49: very low evidence; 3.5-5.49: low evidence; 5.5-6.99: moderate evidence; ≥7: high evidence

**NutriGrade scoring system for SRs without MA of cohort studies**

1. Risk of bias/ study quality/ study limitations **(2 P)**
   1. No information available (0 P)
   2. Risk of bias (2 P)
      1. Ascertainment of exposure^1^
      2. Adjusted basic & outcome relevant model^1^
      3. Assessment of outcome^1^
      4. Adequacy of follow-up duration^1^
   3. Study quality (2 P)^2^
2. Precision **(1 P)**
   1. <500 Events or <2000 participants events (0 P)
   2. ≥500 Events or ≥2000 participants events (1 P)
3. Heterogeneity **(1 P)**
   1. >1/3 of included studies have an inconsistent result (i.e. point estimates and/or 95% CI did not overlap between studies) (0 P)
   2. ≥2/3 of included studies have a consistent result (i.e. point estimates and/or 95% CI did overlap between studies) (1 P)
4. Directness **(1 P)**
   1. Differences in population; differences in intervention; surrogate markers; network meta-analysis (0 P)
   2. No important differences in population or intervention; hard clinical outcome (1 P)
5. Funding bias **(1 P)**
   1. Industry funding OR conflict of interest (0 P)
   2. Private institutions, foundations, non-governmental organizations (0.5 P)
   3. Academic institutions, research institutions (1 P)
6. Effect size **(2 P)**
   1. No effect for >1/3 of included studies (0 P)
   2. Moderate effect size for ≥2/3 of included studies (1 P)
   3. Large effect size for ≥2/3 of included studies (2 P)

**Overall score**^3^

95% CI: confidence intervals; P: point(s); RR: risk ratio.

^1^ ≥2/3 of studies low risk of bias = 0.5 P; >1/3 of studies high risk of bias OR not assessed = 0 P; unclear risk of bias = 0.25 P)

^2^ cut-off for different quality scale (≥3/4 of overall score= 2 P; ≥1/2 of overall score= 1 P; <1/2 of overall score= 0 P); i.e. **Newcastle-Ottawa Scale** (mean): ≥7= 2 P; 4-6.9= 1 P; 0-3.9= 0 P;

^3^ 0-2.99: very low evidence; 3-4.49: low evidence; 4.5-5.99: moderate evidence; ≥6: high evidence
