# Supplementary material for: Protein intake and body weight, fat mass and waist circumference: an umbrella review of systematic reviews for the evidence-based guideline on protein intake of the German Nutrition Society
Source: Eur J Nutr. 2023 Oct 4;63(1):3–32. doi: 10.1007/s00394-023-03220-x (PMC10799103; doi:10.1007/s00394-023-03220-x)
Supplement: Supplementary file 4 — Supplementary file4 (DOCX 37 KB) [file 394_2023_3220_MOESM4_ESM.docx]

Supplementary Material 4. List of excluded articles with rationale for exclusion.

| **Reference** | **Rationale for exclusion** |
| --- | --- |
| Abargouei AS, Janghorbani M, Salehi-Marzijarani M, Esmaillzadeh A: Effect of dairy consumption on weight and body composition in adults: a systematic review and meta-analysis of randomized controlled clinical trials. Int J Obes 2012;36:1485–1493. | Irrelevant exposure |
| Abedini M, Falahi E, Roosta S: Dairy product consumption and the metabolic syndrome. Diabetes Metab Syndr 2015;9:34–37. | Irrelevant exposure |
| Akhlaghi M, Zare M, Nouripour F: Effect of soy and soy isoflavones on obesity-related anthropometric measures: a systematic review and meta-analysis of randomized controlled clinical trials. Adv Nutr 2017;8:705–717. | Irrelevant exposure |
| Ali Redha A, Valizadenia H, Siddiqui SA, Maqsood S: A state-of-art review on camel milk proteins as an emerging source of bioactive peptides with diverse nutraceutical properties. Food Chem 2022;373:131444. | Irrelevant exposure |
| Amirani E, Milajerdi A, Reiner Ž, Mirzaei H, Mansournia MA, Asemi Z: Effects of whey protein on glycemic control and serum lipoproteins in patients with metabolic syndrome and related conditions: a systematic review and meta-analysis of randomized controlled clinical trials. Lipids Health Dis 2020;19:1–18. | Irrelevant outcome |
| Anton SD, Hida A, Heekin K, Sowalsky K, Karabetian C, Mutchie H, Leeuwenburgh C, Manini TM, Barnett TE: Effects of popular diets without specific calorie targets on weight loss outcomes: systematic review of findings from clinical trials. Nutrients 2017;9:822. | Irrelevant exposure |
| Armendariz-Anguiano AL, Jiménez-Cruz A, Bacardi-Gascón M, Pérez-Morales ME: Efectividad del uso de suplementos de proteína en entrenamientos de fuerza: Revisión sistemática. Arch Latinoam Nutr 2010;60:113–118. | Irrelevant language |
| Astrup A, Chaput JP, Gilbert JA, Lorenzen JK: Dairy beverages and energy balance. Physiol Behav 2010;100:67–75. | Irrelevant study type |
| Astrup A: Protein, carbs and fats in personalized weight control – efficacy, effectiveness and safety: Abstracts of the 21th European Congress on Obesity (ECO2014), May 28-31, 2014, Sofia, Bulgaria. Obes Facts 2014;7 Suppl 1:3. | Only abstract available |
| Bauer J, Biolo G, Cederholm T, Cesari M, Cruz-Jentoft AJ, Morley JE, Phillips S, Sieber C, Stehle P, Teta D, Visvanathan R, Volpi E, Boirie Y: Evidence-based recommendations for optimal dietary protein intake in older people: a position paper from the PROT-AGE Study Group. J Am Med Dir Assoc 2013;14:542–559. | Irrelevant study type |
| Blachier F, Beaumont M, Portune KJ, Steuer N, Lan A, Audebert M, Khodorova N, Andriamihaja M, Airinei G, Benamouzig R, Davila A-M, Armand L, Rampelli S, Brigidi P, Tomé D, Claus SP, Sanz Y: High-protein diets for weight management: interactions with the intestinal microbiota and consequences for gut health. A position paper by the my new gut study group. Clin Nutr 2019;38:1012–1022. | Irrelevant study type |
| Blanco Mejia S, Kendall CWC, Viguiliouk E, Augustin LS, Ha V, Cozma AI, Mirrahimi A, Maroleanu A, Chiavaroli L, Leiter LA, Souza RJ de, Jenkins DJA, Sievenpiper JL: Effect of tree nuts on metabolic syndrome criteria: a systematic review and meta-analysis of randomised controlled trials. BMJ Open 2014;4:e004660. | Irrelevant exposure |
| Body J-J: Dairy products: facts and fiction: World Congress on osteoporosis, osteoarthritis and musculoskeletal diseases (WCO-IOF-ESCEO 2015): satellite symposia abstracts. Osteoporos Int 2015;26, Suppl 1:383. | Only abstract available |
| Booth AO, Huggins CE, Wattanapenpaiboon N, Nowson CA: Effect of calcium supplement and dairy food interventions on body weight: a meta-­analysis: Conference Abstracts, Posters. Australasian Med J 2011;4:752. | Only abstract available |
| Bosse JD, Dixon BM: Dietary protein in weight management: a review proposing protein spread and change theories. Nutr Metab 2012;9:81. | Irrelevant outcome |
| Bray GA, Siri-Tarino PW: The role of macronutrient content in the diet for weight management. Endocrinol Metab Clin North Am 2016;45:581–604. | Irrelevant study type |
| Camargo LdR, Doneda D, Oliveira VR: Whey protein ingestion in elderly diet and the association with physical, performance and clinical outcomes. Exp Gerontol 2020;137:110936. | Critically low AMSTAR 2 rating |
| Cawood AL, Elia M, Stratton RJ: Systematic review and meta-analysis of the effects of high protein oral nutritional supplements. Ageing Res Rev 2012;11:278–296. | Irrelevant population |
| Chen G-C, Szeto IMY, Chen L-H, Han S-F, Li Y-J, van Hekezen R, Qin L-Q: Dairy products consumption and metabolic syndrome in adults: systematic review and meta-analysis of observational studies. Sci Rep 2015;5:14606. | Irrelevant exposure |
| Chen M, Pan A, Malik VS, Hu FB: Effects of dairy intake on body weight and fat: a meta-analysis of randomized controlled trials. Am J Clin Nutr 2012;96:735–747. | Irrelevant exposure |
| Cheng H, Kong J, Underwood C, Petocz P, Hirani V, Dawson B, O'Leary F: Systematic review and meta-analysis of the effect of protein and amino acid supplements in older adults with acute or chronic conditions. Br J Nutr 2018;119:527–542. | Irrelevant outcome |
| Clifton PM, Bastiaans K, Keogh JB: High protein diets decrease total and abdominal fat and improve CVD risk profile in overweight and obese men and women with elevated triacylglycerol. Nutr Metab Cardiovasc Dis 2009;19:548–554. | Irrelevant study type |
| Coelho-Júnior HJ, Milano-Teixeira L, Rodrigues B, Bacurau R, Marzetti E, Uchida M: Relative protein intake and physical function in older adults: a systematic review and meta-analysis of observational studies. Nutrients 2018;10:1330. | Irrelevant outcome |
| Coelho-Júnior HJ, Rodrigues B, Uchida M, Marzetti E: Low protein intake is associated with frailty in older adults: a systematic review and meta-analysis of observational studies. Nutrients 2018;10:1334. | Irrelevant outcome |
| Cope MB, Erdman JW, Allison DB: The potential role of soyfoods in weight and adiposity reduction: an evidence-based review. Obes Rev 2008;9:219–235. | Irrelevant exposure |
| Da Silva MS, Rudkowska I: Dairy products on metabolic health: current research and clinical implications. Maturitas 2014;77:221–228. | Irrelevant exposure |
| Dewansingh P, Reckman GAR, Mijlius CF, Krijnen WP, van der Schans CP, Jager-Wittenaar H, van den Heuvel EGHM: Protein, Calcium, Vitamin D Intake and 25(OH)D Status in Normal Weight, Overweight, and Obese Older Adults: A Systematic Review and Meta-Analysis. Front Nutr 2021;8:1–15. | Relevant diet-disease-relationship not investigated |
| Di Rosa C, Lattanzi G, Taylor SF, Manfrini S, Khazrai YM: Very low calorie ketogenic diets in overweight and obesity treatment: effects on anthropometric parameters, body composition, satiety, lipid profile and microbiota. Obes Res Clin Pract 2020;14:491–503. | Irrelevant exposure |
| Donaldson AIC, Smith TO, Alder S, Johnstone AM, de Roos B, Aucott LS, Gordon AL, Myint PK: Effect of non-meat, high protein supplementation on quality of life and clinical outcomes for older people living in care homes: systematic review and meta-analysis. Age Ageing 2017;46:ii17. | Only abstract available |
| Dror DK: Dairy consumption and pre-school, school-age and adolescent obesity in developed countries: a systematic review and meta-analysis. Obes Rev 2014;15:516–527. | Irrelevant exposure |
| Eales J, Lenoir-Wijnkoop I, King S, Wood H, Kok FJ, Shamir R, Prentice A, Edwards M, Glanville J, Atkinson RL: Is consuming yoghurt associated with weight management outcomes? Results from a systematic review. Int J Obes 2016;40:731–746. | Irrelevant exposure |
| Fernandez MA, Panahi S, Daniel N, Tremblay A, Marette A: Yogurt and cardiometabolic diseases: a critical review of potential mechanisms. Adv Nutr 2017;8:812–829. | Irrelevant exposure |
| Fernandez MA, Picard-Deland E, Daniel N, Marette A: Yaourt et santé revue des données récentes. Cah de Nutr et de Diet 2017;52, Suppl:S48–S57. | Irrelevant language |
| Ferreira H, Vasconcelos M, Gil AM, Pinto E: Benefits of pulse consumption on metabolism and health: a systematic review of randomized controlled trials. Crit Rev Food Sci Nutr 2020:1–12. | Irrelevant exposure |
| Finger D, Goltz FR, Umpierre D, Meyer E, Rosa LHT, Schneider CD: Effects of protein supplementation in older adults undergoing resistance training: a systematic review and meta-analysis. Sports Med 2015;45:245–255. | Irrelevant outcome |
| Fu Z, Almahmassani H, Chung M, Cook C: A systematic review of randomized clinical trials examining effects of breakfast composition on weight outcomes among children and adolescents. FASEB J 2017;31, Suppl 1:641.7–641.7. | Only abstract available |
| Gargallo Fernández M, Quiles Izquierdo J, Basulto Marset J, Breton Lesmes I, Formiguera Sala X, Salas-Salvadó J: Evidence-based nutritional recommendations for the prevention and treatment of overweight and obesity in adults (FESNAD-SEEDO consensus document). The role of diet in obesity prevention (II/III). Nutr Hosp 2012;27:800–832. | Irrelevant language |
| Geng T, Qi L, Huang T: Effects of dairy products consumption on body weight and body composition among adults: an updated meta-analysis of 37 randomized control trials. Mol Nutr Food Res 2018;62. | Irrelevant exposure |
| Goisser S, Kiesswetter E, Schoene D, Torbahn G, Bauer JM: Dietary weight-loss interventions for the management of obesity in older adults. Rev Endocr Metab Disord 2020;21:355–368. | Irrelevant study type |
| Gonzalez-Campoy JM, St Jeor ST, Castorino K, Ebrahim A, Hurley D, Jovanovic L, Mechanick JI, Petak SM, Yu Y-H, Harris KA, Kris-Etherton P, Kushner R, Molini-Blandford M, Nguyen QT, Plodkowski R, Sarwer DB, Thomas KT: Clinical practice guidelines for healthy eating for the prevention and treatment of metabolic and endocrine diseases in adults: cosponsored by the American Association of Clinical Endocrinologists/the American College of Endocrinology and the Obesity Society. Endocr Pract 2013;19 Suppl 3:1–82. | Irrelevant study type |
| Gosby AK, Conigrave AD, Raubenheimer D, Simpson SJ: Protein leverage and energy intake. Obes Rev 2014;15:183–191. | Relevant diet-disease-relationship not investigated |
| Gow ML, Ho M, Burrows T, Baur LA, Stewart L, Hutchesson M, Cowell CT, Collins CE, Garnett SP: Macronutrient distribution of the diet–impact on weight and cardiometabolic outcomes in overweight and obese children and adolescents: a systematic review. ANZOS 2013 abstracts. Obes Res Clin Pract 2013;7:e73–e74. | Only abstract available |
| Hein D, Gregory P, Abe A, Wilson A, Risoldi Cochrane Z: The impact of whey protein supplementation on muscle strength and body composition: a systematic review and metaanalysis. Pharmacotherapy 2013;33:e267. | Only abstract available |
| Helms ER, Zinn C, Rowlands DS, Brown SR: A systematic review of dietary protein during caloric restriction in resistance trained lean athletes: a case for higher intakes. Int J Sport Nutr Exerc Metab 2014;24:127–138. | Irrelevant population |
| Hession M, Rolland C, Kulkarni U, Wise A, Broom J: Systematic review of randomized controlled trials of low-carbohydrate vs. low-fat/low-calorie diets in the management of obesity and its comorbidities. Obes Rev 2009;10:36–50. | Irrelevant exposure |
| Hong JY, Lee JS, Woo HW, Om AS, Kwock CK, Kim MK: Meta-analysis of randomized controlled trials on calcium supplements and dairy products for changes in body weight and obesity indices. Int J Food Sci Nutr 2021;72:615–631. | Irrelevant exposure |
| Hudson JL, Bergia RE, Campbell WW: Effects of consuming protein-rich supplements between or with meals on changes in body composition with resistance training: A systematic review of randomized controlled trials. FASEB Journal 2017; 31:1, Suppl 1. | Only abstract available |
| Hudson JL, Bergia RE, Campbell WW: Effects of protein supplements consumed with meals, versus between meals, on resistance training-induced body composition changes in adults: a systematic review. Nutr Rev 2018;76:461–468. | Other |
| Hudson JL, Wang Y, Bergia Iii RE, Campbell WW: Protein intake greater than the RDA differentially influences whole-body lean mass responses to purposeful catabolic and anabolic stressors: a systematic review and meta-analysis. Adv Nutr 2019;11:34. | Irrelevant exposure |
| Hudson JL, Wang Y, Bergia Iii RE, Campbell WW: Protein intake greater than the RDA differentially influences whole-body lean mass responses to purposeful catabolic and anabolic stressors: a systematic review and meta-analysis. Adv Nutr 2019;11:34. | Irrelevant outcome |
| Jäger R, Kerksick CM, Campbell BI, Cribb PJ, Wells SD, Skwiat TM, Purpura M, Ziegenfuss TN, Ferrando AA, Arent SM, Smith-Ryan AE, Stout JR, Arciero PJ, Ormsbee MJ, Taylor LW, Wilborn CD, Kalman DS, Kreider RB, Willoughby DS, Hoffman JR, Krzykowski JL, Antonio J: International Society of Sports Nutrition Position Stand: protein and exercise. J Int Soc Sports Nutr 2017;14. | Irrelevant study type |
| Jin S, Je Y: Dairy consumption and risk of metabolic syndrome: Results from korean population and meta-analysis. Nutrients 2021;13:1574. | Irrelevant exposure |
| Kaiser KA, Jeansonne MM, Brown AW: The effect of yogurt on human body weight: A systematic review and meta-analysis of randomized experiments. FASEB Journal 2016;30. | Only abstract available |
| Kataoka J, Tassone EC, Misso M, Joham AE, Stener-Victorin E, Teede H, Moran LJ: Weight management interventions in women with and without PCOS: a systematic review. Nutrients 2017;9:996. | Irrelevant exposure |
| Kern HJ, Mitmesser SH: Role of nutrients in metabolic health: Updates in 2016. FASEB Journal 2017; 31:1, Suppl 1. | Only abstract available |
| Kim JE, Sands L, Slebodnik M, Connor LO, Campbell W: Effects of high-protein weight loss diets on fat-free mass changes in older adults: A systematic review. FASEB Journal 2014; 28:1, Suppl 1. | Only abstract available |
| Kim JE: Impact of Whey Protein‐Rich Higher‐Protein Diet on Body Weight and Composition Management in Middle‐Aged and Older Adults. Ann Nutr Metab 2019;75, Suppl 3:19–20. | Only abstract available |
| Kim SJ, Souza RJ de, Choo VL, Ha V, Cozma AI, Chiavaroli L, Mirrahimi A, Blanco Mejia S, Di Buono M, Bernstein AM, Leiter LA, Kris-Etherton PM, Vuksan V, Beyene J, Kendall CW, Jenkins DJ, Sievenpiper JL: Effects of dietary pulse consumption on body weight: a systematic review and meta-analysis of randomized controlled trials. Am J Clin Nutr 2016;103:1213–1223. | Irrelevant exposure |
| Kim Y, Keogh J, Clifton PM: Nuts and cardio-metabolic disease: a review of meta-analyses. Nutrients 2018;10:1935. | Irrelevant study type |
| Kirschner MA: Management of Obesity. CRMR 2008;4:122–130. | Irrelevant study type |
| Klonizakis M, Bugg A, Hunt B, Theodoridis X, Bogdanos DP, Grammatikopoulou MG: Assessing the physiological effects of traditional regional diets targeting the prevention of cardiovascular disease: A systematic review of randomized controlled trials implementing mediterranean, New Nordic, Japanese, Atlantic, Persian and Mexican dietary interventions. Nutrients 2021;13:3034. | Irrelevant exposure |
| Kongerslev Thorning T, Raben A, Tholstrup T, Soedamah-Muthu SS, Givens I, Astrup A: Milk and dairy products: good or bad for human health? An assessment of the totality of scientific evidence. Food Nutr Res 2016;60:32527. | Irrelevant study type |
| Kouris-Blazos A, Belski R: Health benefits of legumes and pulses with a focus on Australian sweet lupins. Asia Pac J Clin Nutr 2016;25:1–17. | Relevant diet-disease-relationship not investigated |
| Kromhout D, Spaaij CJK, Goede J de, Weggemans RM: The 2015 Dutch food-based dietary guidelines. Eur J Clin Nutr 2016;70:869–878. | Relevant diet-disease-relationship not investigated |
| Lancha AH, Zanella R, Tanabe SGO, Andriamihaja M, Blachier F: Dietary protein supplementation in the elderly for limiting muscle mass loss. Amino acids 2017;49:33–47. | Irrelevant study type |
| Lanou AJ, Barnard ND: Dairy and weight loss hypothesis: an evaluation of the clinical trials. Nutr Rev 2008;66:272–279. | Irrelevant exposure |
| Lee M, Lee H, Kim J: Dairy food consumption is associated with a lower risk of the metabolic syndrome and its components: a systematic review and meta-analysis. Br J Nutr 2018;120:373–384. | Irrelevant exposure |
| Lee-Bravatti MA, Wang J, Avendano EE, King L, Johnson EJ, Raman G: Almond consumption and risk factors for cardiovascular disease: a systematic review and meta-analysis of randomized controlled trials. Adv Nutr 2019;10:1076–1088. | Irrelevant exposure |
| Leidy HJ, Clifton PM, Astrup A, Wycherley TP, Westerterp-Plantenga MS, Luscombe-Marsh ND, Woods SC, Mattes RD: The role of protein in weight loss and maintenance. Am J Clin Nutr 2015;101, Suppl:1320S–1329S. | Irrelevant study type |
| Li J, Partridge J, Sulo S.: Nutrition interventions positively impact health outcomes of community-based adults: A systematic review: 22nd International Abstracts Book. PHP 248. Value in Health 2017;20:A58–A59. | Only abstract available |
| Lombardo M, Bellia C, Moletto C, Aulisa G, Padua E, Della-Morte D, Caprio M, Bellia A: Effects of quality and quantity of protein intake for type 2 diabetes mellitus prevention and metabolic control. Curr Nutr Rep 2020;9:329–337. | Irrelevant outcome |
| Louie JC, Flood VM, Hector DJ, Rangan AM, Gill TP: Dairy consumption and overweight and obesity: a systematic review of prospective cohort studies. Obes Rev 2011;12:e582–e592. | Irrelevant exposure |
| Machado de Souza RG, Machado Schincaglia R, Pimentel GD, Mota JF: Nuts and human health outcomes: a systematic review. Nutrients 2017;9:1311. | Irrelevant exposure |
| Magkos F: The role of dietary protein in obesity. Rev Endocr Metab Disord 2020;21:329–340. | Irrelevant study type |
| Messina M, Lynch H, Dickinson JM, Reed KE: No difference between the effects of supplementing with soy protein versus animal protein on gains in muscle mass and strength in response to resistance exercise. Int J Sport Nutr Exerc Metab 2018;28:674–685. | Irrelevant outcome |
| Mu Y, Kou T, Wei B, Lu X, Liu J, Tian H, Zhang W, Liu B, Li H, Cui W, Wang Q: Soy products ameliorate obesity-related anthropometric indicators in overweight or obese Asian and non-menopausal women: a meta-analysis of randomized controlled trials. Nutrients 2019;11:2790. | Irrelevant exposure |
| Naclerio F, Larumbe-Zabala E: Effects of whey protein alone or as part of a multi-ingredient formulation on strength, fat-free mass, or lean body mass in resistance-trained individuals: a meta-analysis. Sports Med 2016;46:125–137. | Irrelevant outcome |
| O‘Bryan KR, Doering TM, Morton RW, Coffey VG, Phillips SM, Cox GR: Do multi-ingredient protein supplements augment resistance training-induced gains in skeletal muscle mass and strength? A systematic review and meta-analysis of 35 trials. Br J Sports Med 2020;54:573–581. | Irrelevant exposure |
| Oktaviana J, Zanker J, Vogrin S, Duque G: The effect of protein supplements on functional frailty in older persons: a systematic review and meta-analysis. Arch Gerontol Geriatr 2020;86:103938. | Irrelevant outcome |
| Pal S, Radavelli-Bagatini S: The effects of whey protein on cardiometabolic risk factors. Obes Rev 2013;14:324–343. | Irrelevant study type |
| Pedersen AN, Cederholm T: Health effects of protein intake in healthy elderly populations: a systematic literature review. Food Nutr Res 2014;58. | Irrelevant outcome |
| Poscia A, Milovanovic S, La Milia DI, Duplaga M, Grysztar M, Landi F, Moscato U, Magnavita N, Collamati A, Ricciardi W: Effectiveness of nutritional interventions addressed to elderly persons: umbrella systematic review with meta-analysis. Eur J Public Health 2017;28:275–283. | Irrelevant study type |
| Prentice AM: Dairy products in global public health. Am J Clin Nutr 2014;99, Suppl:1212S–1216S. | Irrelevant exposure |
| Reinders I, Volkert D, Groot LCPGM de, Beck AM, Feldblum I, Jobse I, Neelemaat F, van der Schueren MAE de, Shahar DR, Smeets ETHC, Tieland M, Twisk JWR, Wijnhoven HAH, Visser M: Effectiveness of nutritional interventions in older adults at risk of malnutrition across different health care settings: pooled analyses of individual participant data from nine randomized controlled trials. Clin Nutr 2019;38:1797–1806. | Irrelevant exposure |
| Robertson C, Aceves Martins M, Stewart F, Cooper D, Avenell A, Rebalance Project: A systematic review of long-term randomised controlled trial evidence of interventions for weight management in people with BMI ≥35 kg/m2: The REBALANCE Project. Obes Facts 2019;12, Suppl 1:178. | Only abstract available |
| Ruscica M, Pavanello C, Gandini S, Gomaraschi M, Vitali C, Macchi C, Aiello G, Bosisio R, Calabresi L, Arnoldi A, Sirtori CR, Magni P: Effect of soy on metabolic syndrome and cardiovascular risk factors: evidence from a randomized controlled trial. Nutr Metab Cardiovasc Dis 2017;27:e35. | Only abstract available |
| Salas-Salvadó J: Dairy product consumption and risk of cardiovascular diseases. Ann Nutr Metab 2017;71, Suppl 2:270–271. | Only abstract available |
| Santos FL, Esteves SS, da Costa Pereira A, Yancy WS, Nunes JPL: Systematic review and meta-analysis of clinical trials of the effects of low carbohydrate diets on cardiovascular risk factors. Obes Rev 2012;13:1048–1066. | Irrelevant exposure |
| Sayon-Orea C, Martínez-González MA, Ruiz-Canela M, Bes-Rastrollo M: Associations between yogurt consumption and weight gain and risk of obesity and metabolic syndrome: a systematic review. Adv Nutr 2017;8, Suppl:146S–154S. | Irrelevant exposure |
| Schneider BC, Dumith SC, Orlandi SP, Assunção MCF: Diet and body fat in adolescence and early adulthood: a systematic review of longitudinal studies. Cien Saude Colet 2017;22:1539–1552. | Irrelevant population |
| Schwingshackl L, Hoffmann G, Schwedhelm C, Kalle-Uhlmann T, Missbach B, Knüppel S, Boeing H: Consumption of dairy products in relation to changes in anthropometric variables in adult populations: a systematic review and meta-analysis of cohort studies. PLoS One 2016;11:e0157461. | Irrelevant exposure |
| Senior AM, Gosby AK, Lu J, Simpson SJ, Raubenheimer D: Meta-analysis of variance: an illustration comparing the effects of two dietary interventions on variability in weight. Evol Med Public Health 2016;2016:244–255. | Irrelevant exposure |
| Sharma AM: Weight-loss diets only work when you follow them. Evid Based Med 2015;20:103–104. | Irrelevant study type |
| Soedamah-Muthu SS, Goede J de: Dairy consumption and cardiometabolic diseases: systematic review and updated meta-analyses of prospective cohort studies. Curr Nutr Rep 2018;7:171–182. | Irrelevant outcome |
| Stelmach-Mardas M, Walkowiak J: Dietary interventions and changes in cardio-metabolic parameters in metabolically healthy obese subjects: a systematic review with meta-analysis. Nutrients 2016;8:455. | Irrelevant exposure |
| Stonehouse W, Wycherley T, Luscombe-Marsh N, Taylor P, Brinkworth G, Riley M: Dairy intake enhances body weight and composition changes during energy restriction in 18-50-year-old adults - a meta-analysis of randomized controlled trials. Nutrients 2016;8:394. | Irrelevant exposure |
| ten Haaf DSM, Nuijten MAH, Maessen MFH, Horstman AMH, Eijsvogels TMH, Hopman MTE: Effects of protein supplementation on lean body mass, muscle strength, and physical performance in nonfrail community-dwelling older adults: a systematic review and meta-analysis. Am J Clin Nutr 2018;108:1043–1059. | Irrelevant outcome |
| Thompson HJ, McGinley JN, Neil ES, Brick MA: Beneficial effects of common bean on adiposity and lipid metabolism. Nutrients 2017;9:998. | Irrelevant study type |
| Tieland M, Franssen R, Dullemeijer C, van Dronkelaar C, Kyung Kim H, Ispoglou T, Zhu K, Prince RL, van Loon LJC, Groot LCPGM de: The impact of dietary protein or amino acid supplementation on muscle mass and strength in elderly people: individual participant data and meta-analysis of RCT's. J Nutr Health Aging 2017;21:994–1001. | Irrelevant outcome |
| Trabal J, Farran-Codina A: Effects of dietary enrichment with conventional foods on energy and protein intake in older adults: a systematic review. Nutr Rev 2015;73:624–633. | Irrelevant exposure |
| Tucker R: New approaches to dieting and eating: what are they and do they work? J Pharm 2014;292:582–584. | Irrelevant study type |
| Ullah N, Hafeez K, Farooq S, Batool A, Aslam N, Hussain M, Ahmad S: Anti-diabetes and anti-obesity: a meta-analysis of different compounds. Asian Pac J Trop Dis 2016;6:749–756. | Irrelevant study type |
| Wang W, Wu Y, Zhang D: Association of dairy products consumption with risk of obesity in children and adults: a meta-analysis of mainly cross-sectional studies. Ann Epidemiol 2016;26:870–882.e2. | Irrelevant exposure |
| Wirth J, Hillesheim E, Brennan L: The role of protein intake and its timing on body composition and muscle function in healthy adults: a systematic review and meta-analysis of randomized controlled trials. J Nutr 2020;150:1443–1460. | Irrelevant outcome |
| Yang D, Liu Z, Yang H, Jue Y: Acute effects of high-protein versus normal-protein isocaloric meals on satiety and ghrelin. Eur J Nutr 2014;53:493–500. | Irrelevant outcome |
| Zhang X-W, Yang Z, Li M, Li K, Deng Y-Q, Tang Z-Y: Association between dietary protein intake and risk of stroke: A meta-analysis of prospective studies. Int J Cardiol 2016;223:548–551. | Irrelevant outcome |
| Zhang X-M, Zhang Y-B, Chi M-H: Soy protein supplementation reduces clinical indices in type 2 diabetes and metabolic syndrome. Yonsei Med J 2016;57:681–689. | Irrelevant population |
