# Supplementary material for: Protein intake and body weight, fat mass and waist circumference: an umbrella review of systematic reviews for the evidence-based guideline on protein intake of the German Nutrition Society
Source: Eur J Nutr. 2023 Oct 4;63(1):3–32. doi: 10.1007/s00394-023-03220-x (PMC10799103; doi:10.1007/s00394-023-03220-x)
Supplement: Supplementary file 6 — Supplementary file6 (DOCX 35 KB) [file 394_2023_3220_MOESM6_ESM.docx]

**Supplementary Material 6.** Methodological quality assessment of included systematic reviews using AMSTAR 2.

| Assessment  item  Study | 1. Components of PICO included? | 1. Method a priori? Protocol reported? | 1. Comprehensive literature search strategy? | 1. Study selection performed in duplicate? | 1. Data extraction performed in duplicate? | 1. Number of excluded studies and corresponding reasons provided? | 1. Detailed study characteristics provided? | 1. Risk of bias assessed? | 1. Statistical heterogeneity assessed? | 1. Risk of bias considered in the discussion and interpretation? | 1. Discussion of any heterogeneity observed in the results? | 1. Publication bias investigated? | 1. Likely impact of publication bias discussed? | 1. Potential conflicts of interest stated? | Number of critical weaknesses | Number of non-critical weaknesses | Methodological quality |
| --- | --- | --- | --- | --- | --- | --- | --- | --- | --- | --- | --- | --- | --- | --- | --- | --- | --- |
| Damaghi 2022 [46] | Yes | Yes | Yes | Yes | Yes | Yes | Yes | Yes | Yes | No | Yes | Yes | Yes | Yes | 0 | 1 | high |
| Zhang 2022 [50] | Yes | Yes | Yes | Yes | No | Yes | Yes | Yes | Yes | No | No | Yes | Yes | No | 0 | 4 | moderate |
| Hansen 2021 [47] | Yes | Yes | Yes | Yes | No | Yes | Yes | Yes | Yes | Yes | Yes | Yes | Yes | No | 0 | 2 | moderate |
| Mohammadifard 2021 [48] | Yes | Yes | Yes | Yes | No | Yes | Yes | No | Yes | No | Yes | Yes | Yes | Yes | 1 | 2 | low |
| Vogtschmidt 2021 [49] | Yes | Yes | Yes | Yes | Yes | Yes | Yes | Yes | Yes | No | Yes | Yes | Yes | Yes | 0 | 1 | high |
| Blair 2020 [18] | Yes | Yes | Yes | Yes | Yes | Yes | Yes | Yes | No MA | Yes | No | No MA | No MA | Yes | 0 | 1 | high |
| Camargo 2020 | Yes | No | No | No | Yes | Yes | Yes | No | No MA | No | No | No MA | No MA | Yes | 2 | 4 | critically low |
| Lonnie 2020 [19] | Yes | Yes | Yes | Yes | No | Yes | Yes | No | No MA | No | No | No MA | Yes | No | 1 | 4 | low |
| Zhao 2020 [20] | Yes | No | Yes | Yes | Yes | Yes | Yes | Yes | Yes | Yes | Yes | Yes | Yes | Yes | 0 | 1 | high |
| Badely 2019 [21] | Yes | Yes | Yes | Yes | Yes | Yes | Yes | No | Yes | No | No | Yes | Yes | Yes | 1 | 2 | low |
| Donaldson 2019 [22] | Yes | Yes | Yes | Yes | Yes | Yes | Yes | Yes | Yes | No | No | No | No | Yes | 1 | 3 | low |
| Hsu 2019 [23] | Yes | Yes | Yes | No | Yes | Yes | Yes | Yes | Yes | No | No | No | No | Yes | 1 | 4 | low |
| Li 2019 [24] | Yes | Yes | Yes | No | No | Yes | Yes | Yes | Yes | No | Yes | Yes | Yes | No | 0 | 4 | moderate |
| Saboori 2019 [45] | Yes | Yes | Yes | Yes | No | Yes | Yes | Yes | Yes | No | Yes | Yes | Yes | Yes | 0 | 2 | moderate |
| Valenzuela 2019 [25] | Yes | Yes | Yes | No | Yes | Yes | Yes | Yes | Yes | Yes | Yes | Yes | Yes | Yes | 0 | 1 | high |
| van Baak 2019 [26] | Yes | No | No | No | No | Yes | Yes | Yes | Yes | No | Yes | Yes | Yes | Yes | 1 | 4 | low |
| Bergia 2018 [27] | Yes | No | Yes | Yes | Yes | Yes | Yes | Yes | Yes | No | Yes | No | No | Yes | 1 | 3 | low |
| Dewansingh 2018 [28] | Yes | Yes | Yes | Yes | Yes | Yes | Yes | Yes | Yes | No | Yes | Yes | Yes | No | 0 | 2 | moderate |
| Hidayat 2018 [41] | Yes | Yes | Yes | Yes | Yes | Yes | Yes | Yes | Yes | No | Yes | Yes | Yes | Yes | 0 | 1 | high |
| Liao 2018 [29] | Yes | Yes | Yes | Yes | Yes | Yes | Yes | Yes | Yes | Yes | Yes | No | No | Yes | 1 | 1 | low |
| Wirunsawanya 2018 [42] | Yes | Yes | Yes | Yes | Yes | Yes | Yes | Yes | Yes | No | No | Yes | No | No | 0 | 4 | moderate |
| Chalvon-Demersay 2017 [43] | Yes | Yes | Yes | No | No | Yes | Yes | Yes | No MA | Yes | No | No MA | No MA | Yes | 0 | 3 | moderate |
| Liao 2017 [36] | Yes | Yes | Yes | No | Yes | Yes | Yes | Yes | Yes | No | No | No | No | Yes | 1 | 4 | low |
| Kim 2016 [37] | Yes | Yes | Yes | Yes | Yes | Yes | Yes | Yes | Yes | No | No | No | No | Yes | 1 | 3 | low |
| Clifton 2014 [38] | Yes | Yes | Yes | No | Yes | Yes | Yes | Yes | Yes | No | Yes | Yes | Yes | No | 0 | 3 | moderate |
| Johansson 2014 [44] | Yes | No | Yes | Yes | Yes | Yes | Yes | No | Yes | No | Yes | Yes | Yes | Yes | 1 | 2 | low |
| Miller 2014 [40] | Yes | No | Yes | No | Yes | Yes | Yes | No | Yes | No | Yes | Yes | Yes | Yes | 1 | 3 | low |
| Pedersen 2013 [33] | Yes | Yes | Yes | Yes | No | Yes | Yes | Yes | No MA | Yes | No | No MA | No MA | Yes | 0 | 2 | moderate |
| Schwingshackl 2013 [32] | Yes | Yes | Yes | Yes | Yes | Yes | Yes | Yes | Yes | Yes | Yes | Yes | Yes | Yes | 0 | 0 | high |
| Fogelholm 2012 [34] | Yes | No | Yes | Yes | No | Yes | Yes | Yes | No MA | No | No | No MA | No MA | Yes | 0 | 4 | moderate |
| Santesso 2012 [35] | Yes | Yes | Yes | Yes | Yes | Yes | Yes | Yes | Yes | Yes | No | Yes | Yes | Yes | 0 | 1 | high |
| Wycherley 2012 [39] | Yes | No | Yes | Yes | No | Yes | No | Yes | Yes | No | No | No | No | Yes | 1 | 6 | low |
| Lepe 2011 [31] | Yes | No | Yes | No | No | Yes | Yes | No | No MA | No | No | No MA | No MA | No | 1 | 6 | low |
| Summerbell 2009 [30] | Yes | No | Yes | Yes | No | Yes | No | No | No MA | No | No | No MA | No MA | Yes | 1 | 5 | low |

The assessment items are provided as shortened versions. The full questionnaire is provided in Supplementary Material 2. Critical assessment items are underlined.

High rating = no critical weakness with no or one non-critical weakness: the systematic review provides an accurate and comprehensive summary of the results of the available studies that address the question of interest. Moderate rating = no critical weakness with more than one non-critical weakness: the systematic review has more than one weakness but no critical flaws. It may provide an accurate summary of the results of the available studies that were included in the review. Low rating = one critical weakness with or without non-critical weaknesses: the review has a critical flaw and may not provide an accurate and comprehensive summary of the available studies that address the question of interest. Critically low rating = more than one critical weakness with or without non-critical weaknesses: the review has more than one critical flaw and should not be relied on to provide an accurate and comprehensive summary of the available studies. Shea BJ, Reeves BC, Wells G, Thuku M, Hamel C, Moran J, et al. AMSTAR 2: a critical appraisal tool for systematic reviews that include randomised or non-randomised studies of healthcare interventions, or both. BMJ. 2017 Sep;358(j4008):1-8.

AMSTAR 2: A Measurement Tool to Assess Systematic Reviews 2; MA: meta-analysis.
